# Supplementary material for: Six New Phenolic Glycosides from the Seeds of Moringa oleifera Lam. and Their α-Glucosidase Inhibitory Activity
Source: Molecules. 2023 Sep 4;28(17):6426. doi: 10.3390/molecules28176426 (PMC10489651; doi:10.3390/molecules28176426)
Supplement: Supplementary file 1 [file molecules-28-06426-s001.zip › molecules-2534344-supplementary.pdf]

# Six New Phenolic Glycosides from the Seeds of *Moringa oleifera* Lam. and Their *α*-glucosidase Inhibitory Activity

**Lin-Zhen Li [1,2,3,†], Liang Chen [1,4,†], Yang-Li Tu [1,4], Xiang-Jie Dai [1,4], Sheng-Jia Xiao [1,4], Jing-Shan Shi [2,*], Yong-Jun Li [4,*] and Xiao-Sheng Yang [3]**

[1] School of Pharmacy, Guizhou Medical University, Guiyang 550004, China; lilinzhen@gmc.edu.cn (L.-Z.L.); 13624538210@139.com (L.C.); tyltyl715@163.com (Y.-L.T.); daixiangjie2023@163.com (X.-J.D.); xshengjia213@163.com (S.-J.X.)

[2] Key Laboratory of Basic Pharmacology of Ministry of Education, Zunyi Medical University, Zunyi 563000, China

[3] State Key Laboratory of Functions and Applications of Medicinal Plants, Guizhou Medical University, Guiyang 550004, China; yang_xiaosheng@yahoo.com

[4] Engineering Research Center for the Development and Application of Ethnic Medicine and TCM, Ministry of Education, Guizhou Medical University, Guiyang 550004, China

* Correspondence: shijs@zmc.edu.cn (J.-S.S.); liyongjun026@gmc.edu.cn (Y.-J.L.)

† These authors contributed equally to this work.

**Contents**

**Figure S1.** HR-ESI-MS spectrum of compound **1** (postive mode).

**Figure S2.** UV spectrum of compound **1.**

**Figure S3.** IR spectrum of compound **1.**

**Figure S4.** $^1$H-NMR (400MHz, $CD_3OD$) spectrum of compound **1**.

**Figure S5.** $^{13}$C -NMR (100MHz, $CD_3OD$) spectrum of compound **1**.

**Figure S6.** HMQC spectrum of compound **1**.

**Figure S7.** HMBC spectrum of compound **1**.

**Figure S8.** $^1$H-$^1$H COSY spectrum of compound **1**.

**Figure S9.** HR-ESI-MS spectrum of compound **2** (positive mode).

**Figure S10.** UV spectrum of compound **2**.

**Figure S11.** IR spectrum of compound **2**.

**Figure S12.** $^1$H-NMR (600MHz, $CD_3OD$) spectrum of compound **2**.

**Figure S13.** $^{13}$C-NMR (150MHz, $CD_3OD$) spectrum of compound **2**.

**Figure S14.** HMQC spectrum of compound **2**.

**Figure S15.** HMBC spectrum of compound **2**.

**Figure S16.** $^1$H-$^1$H COSY spectrum of compound **2**.

**Figure S17.** NOESY spectrum of compound **2**.

**Figure S18.** HR-ESI-MS spectrum of compound **3** (positive mode).

**Figure S19.** UV spectrum of compound **3**.

**Figure S20.** IR spectrum of compound **3**.

**Figure S21.** $^1$H-NMR (600MHz, $CD_3OD$) spectrum of compound **3**.

**Figure S22.** $^{13}$C-NMR (150MHz, $CD_3OD$) spectrum of compound **3**.

**Figure S23.** HMQC spectrum of compound **3**.

**Figure S24.** HMBC spectrum of compound **3**.

**Figure S25.** $^{1}$H-$^{1}$H COSY spectrum of compound **3**.

**Figure S26.** NOESY spectrum of compound **3**.

**Figure S27.** HR-ESI-MS spectrum of compound **4** (positive mode).

**Figure S28.** UV spectrum of compound **4**.

**Figure S29.** IR spectrum of compound **4**.

**Figure S30.** $^{1}$H-NMR (600MHz, $CD_3OD$) spectrum of compound **4**.

**Figure S31.** $^{13}$C-NMR (150MHz, $CD_3OD$) spectrum of compound **4**.

**Figure S32.** HMQC spectrum of compound **4**.

**Figure S33.** HMBC spectrum of compound **4**.

**Figure S34.** $^{1}$H-$^{1}$H COSY spectrum of compound **4**.

**Figure S35.** HR-ESI-MS spectrum of compound **5** (negative mode).

**Figure S36.** UV spectrum of compound **5**.

**Figure S37.** IR spectrum of compound **5**.

**Figure S38.** $^{1}$H-NMR (600MHz, $CD_3OD$) spectrum of compound **5**.

**Figure S39.** $^{13}$C-NMR (150MHz, $CD_3OD$) spectrum of compound **5**.

**Figure S40.** HMQC spectrum of compound **5**.

**Figure S41.** HMBC spectrum of compound **5**.

**Figure S42.** $^{1}$H-$^{1}$H COSY spectrum of compound **5**.

**Figure S43.** HR-ESI-MS spectrum of compound **6** (positive mode).

**Figure S44.** UV spectrum of compound **6**.

**Figure S45.** IR spectrum of compound **6**.

**Figure S46.** $^{1}$H-NMR (600MHz, $CD_3OD$) spectrum of compound **6**.

**Figure S47.** $^{13}$C-NMR (150MHz, $CD_3OD$) spectrum of compound **6**.

**Figure S48.** HMQC spectrum of compound **6**.

**Figure S49.** HMBC spectrum of compound **6**.

**Figure S50.** $^{1}$H-$^{1}$H COSY spectrum of compound **6**.

**Figure S51.** GC analysis of D-rhamnose, L-rhamnose and compounds **1–6**.

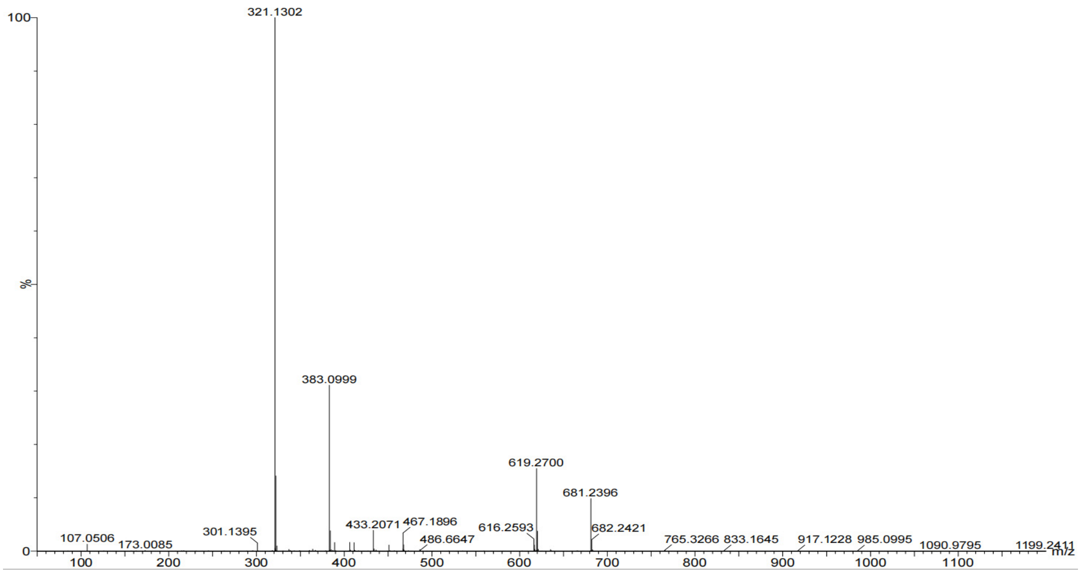


**Figure S1**. HR-ESI-MS spectrum of compound **1** (postive mode).

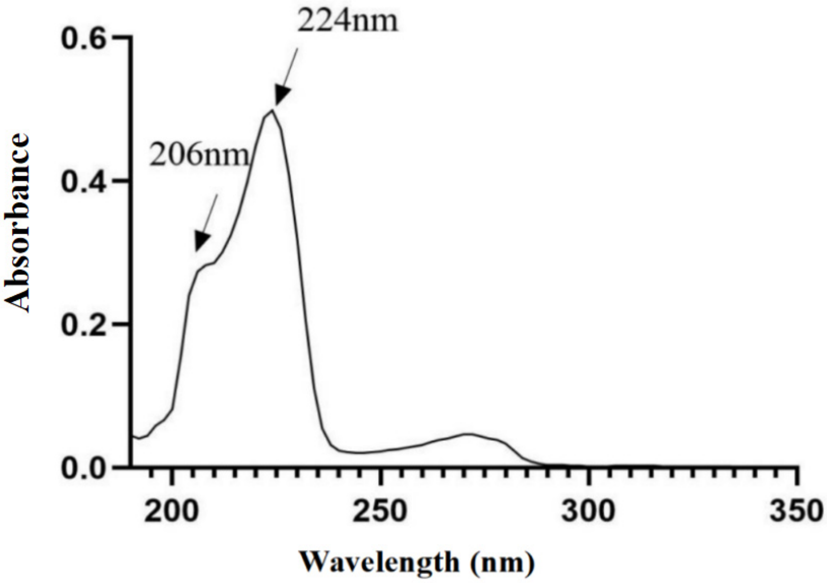


**Figure S2**. UV spectrum of compound **1**.

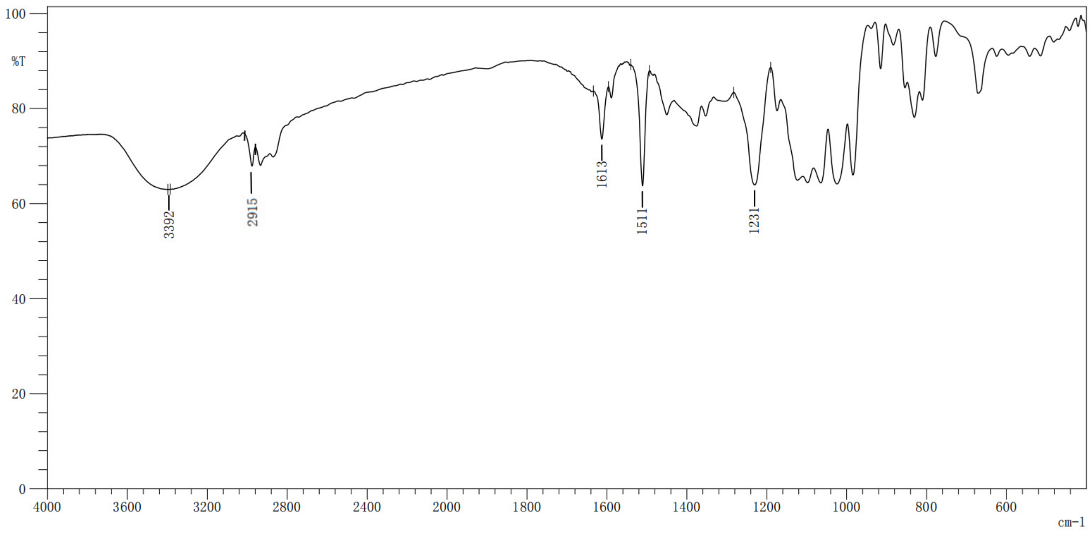


**Figure S3**. IR spectrum of compound **1**.

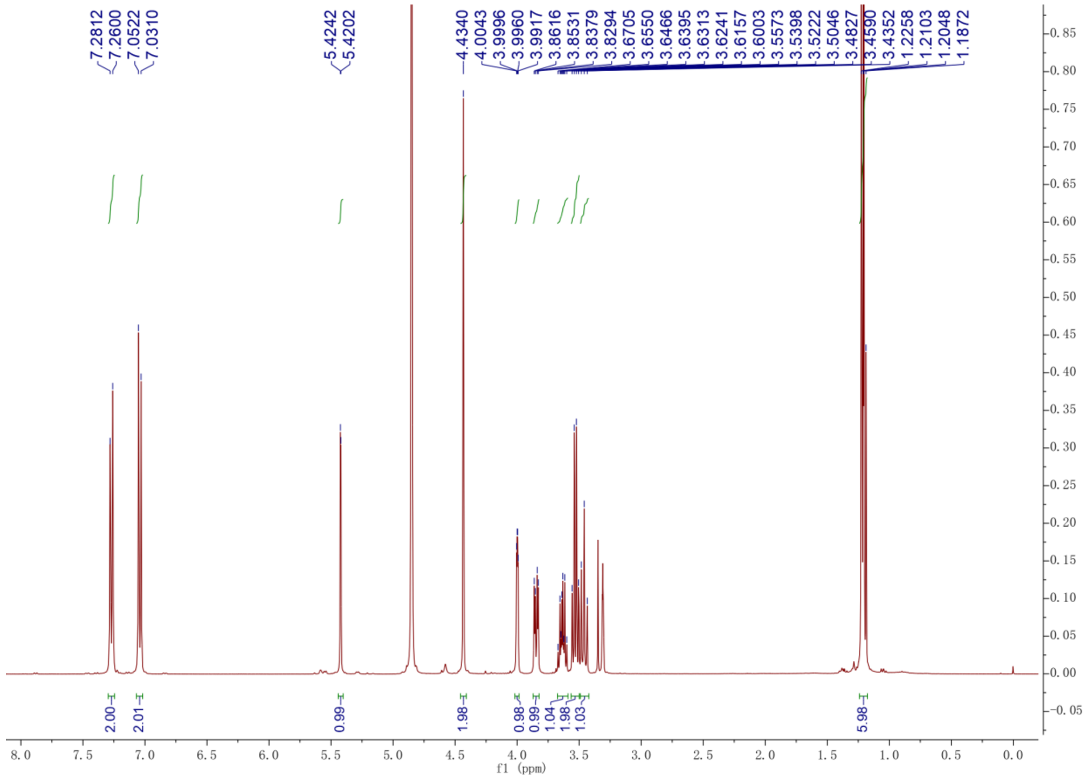


**Figure S4**. $^{1}$H-NMR (400MHz, $CD_3OD$) spectrum of compound **1**.

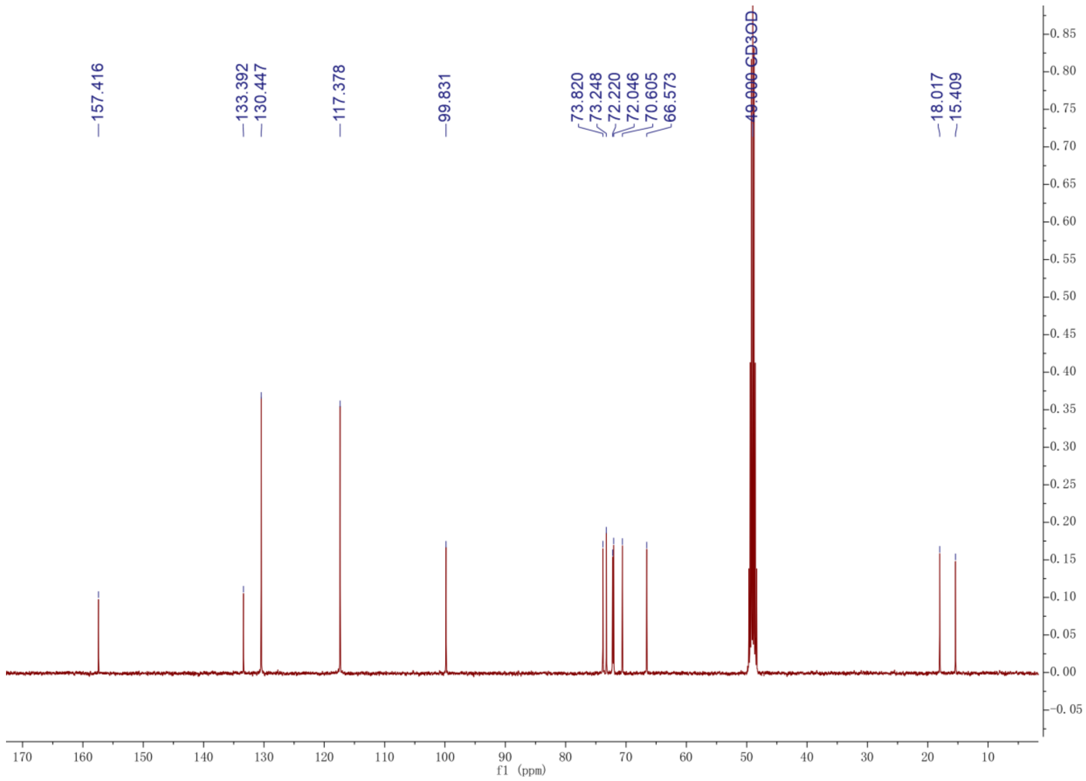


**Figure S5**. $^{13}$C-NMR (100MHz, $CD_3OD$) spectrum of compound **1**.

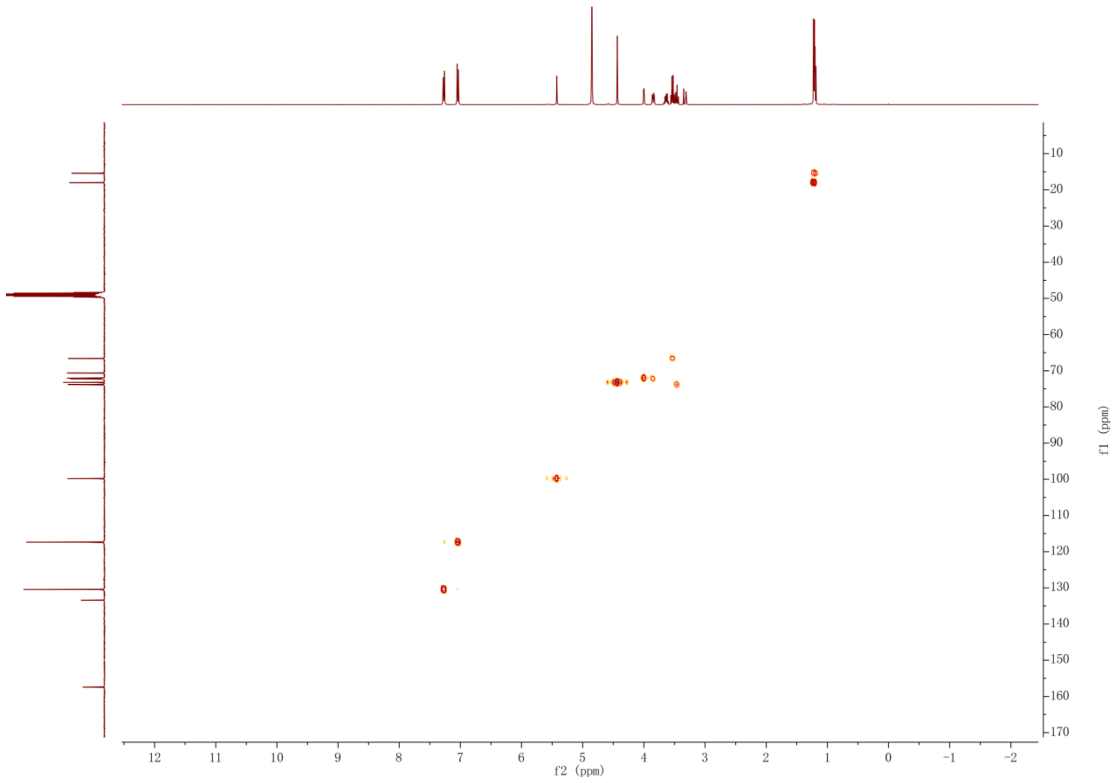


**Figure S6**. HMQC spectrum of compound **1**.

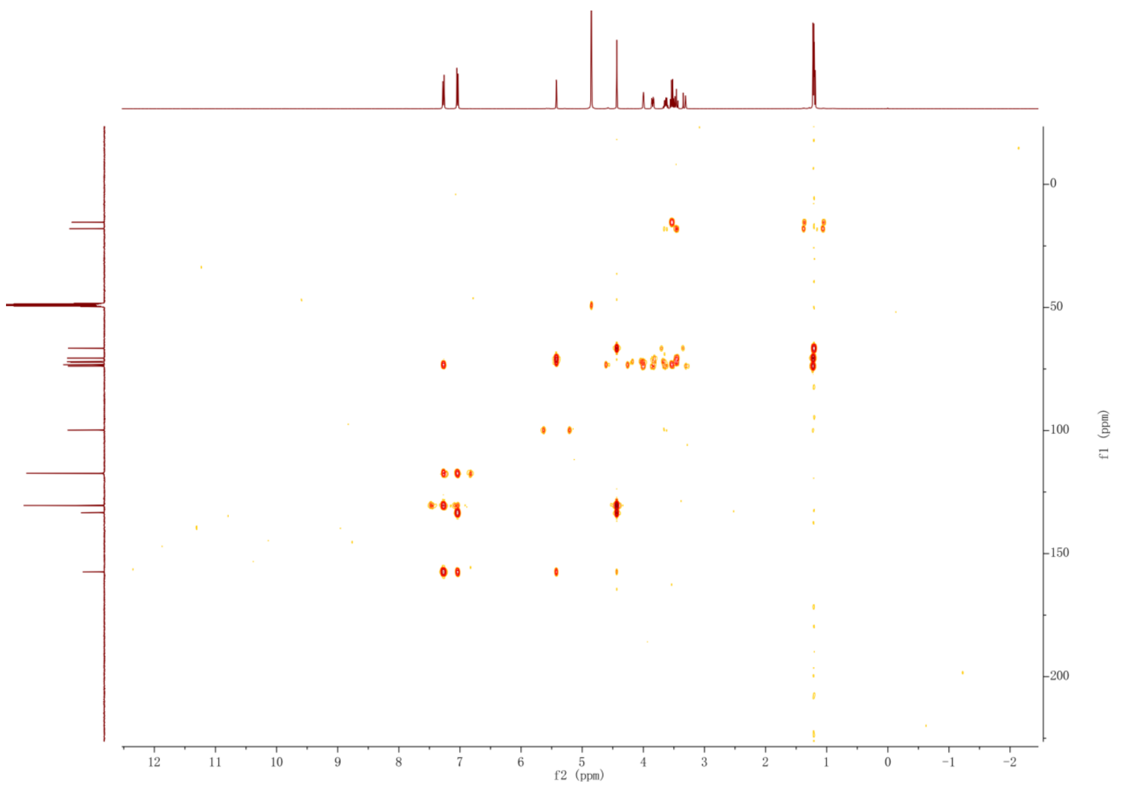


**Figure S7**. HMBC spectrum of compound **1**.

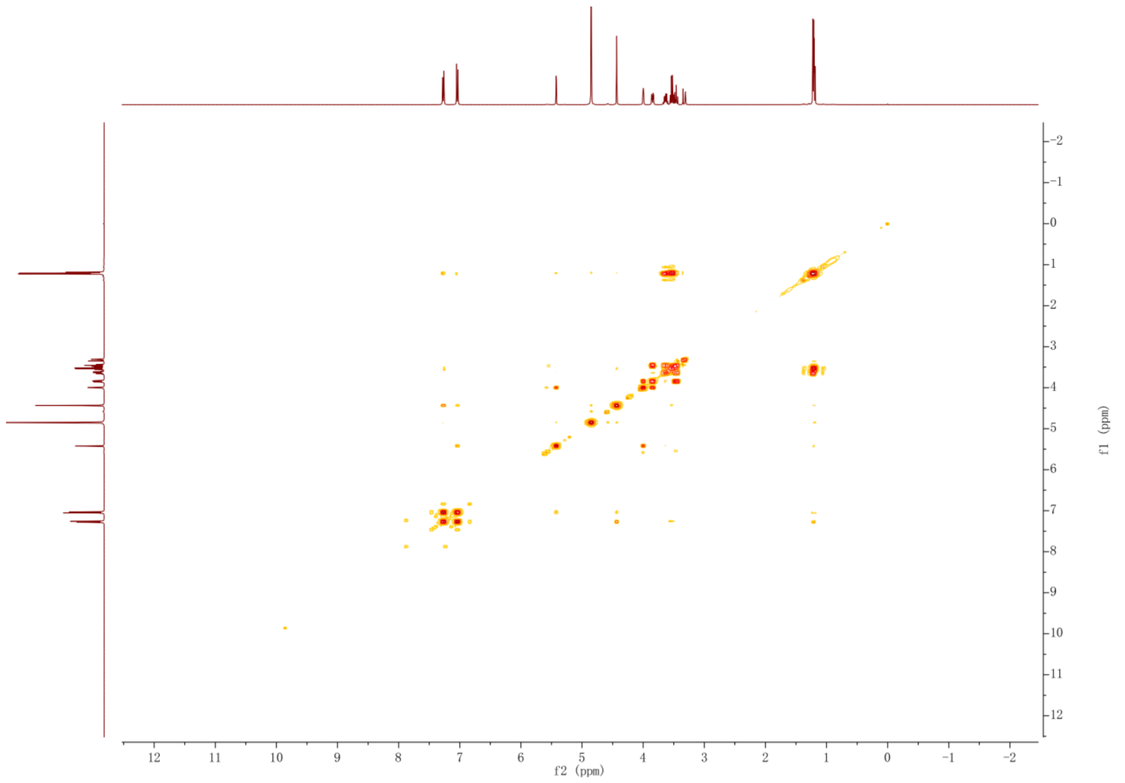


**Figure S8**. $^{1}H$-$^{1}H$ COSY spectrum of compound **1**.

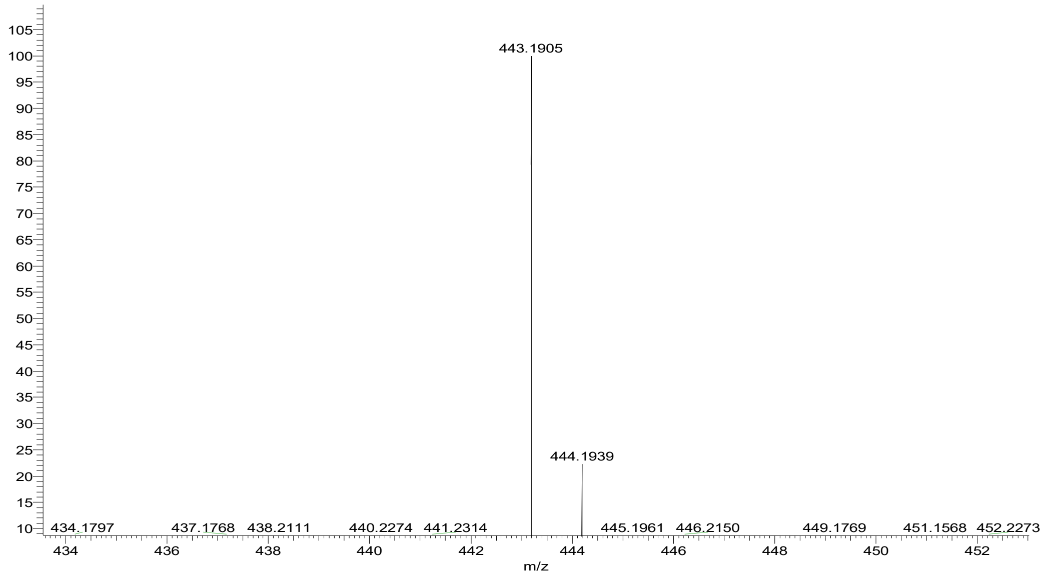


**Figure S9**. HR-ESI-MS spectrum of compound **2** (postive mode).

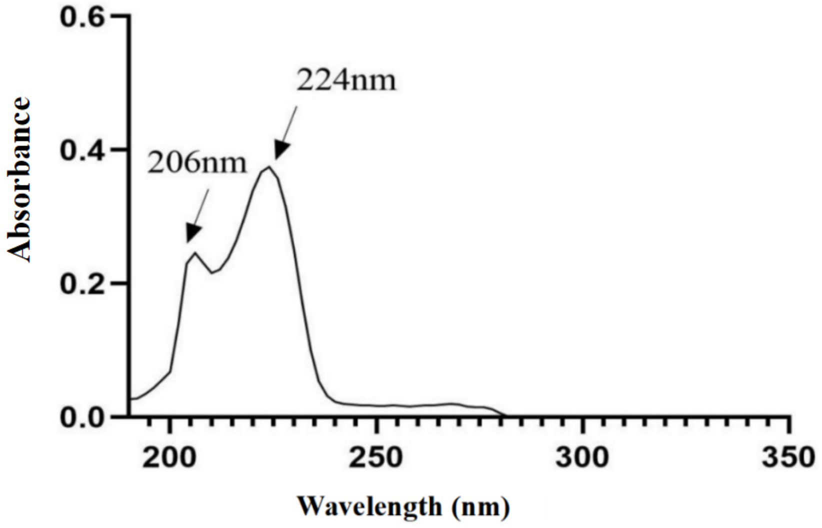


**Figure S10**. UV spectrum of compound **2**.

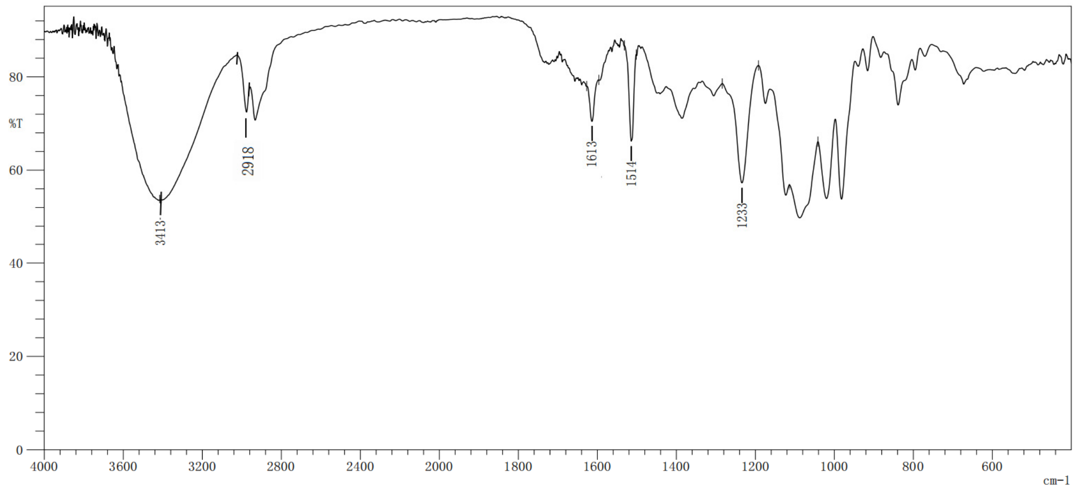


**Figure S11**. IR spectrum of compound **2**.

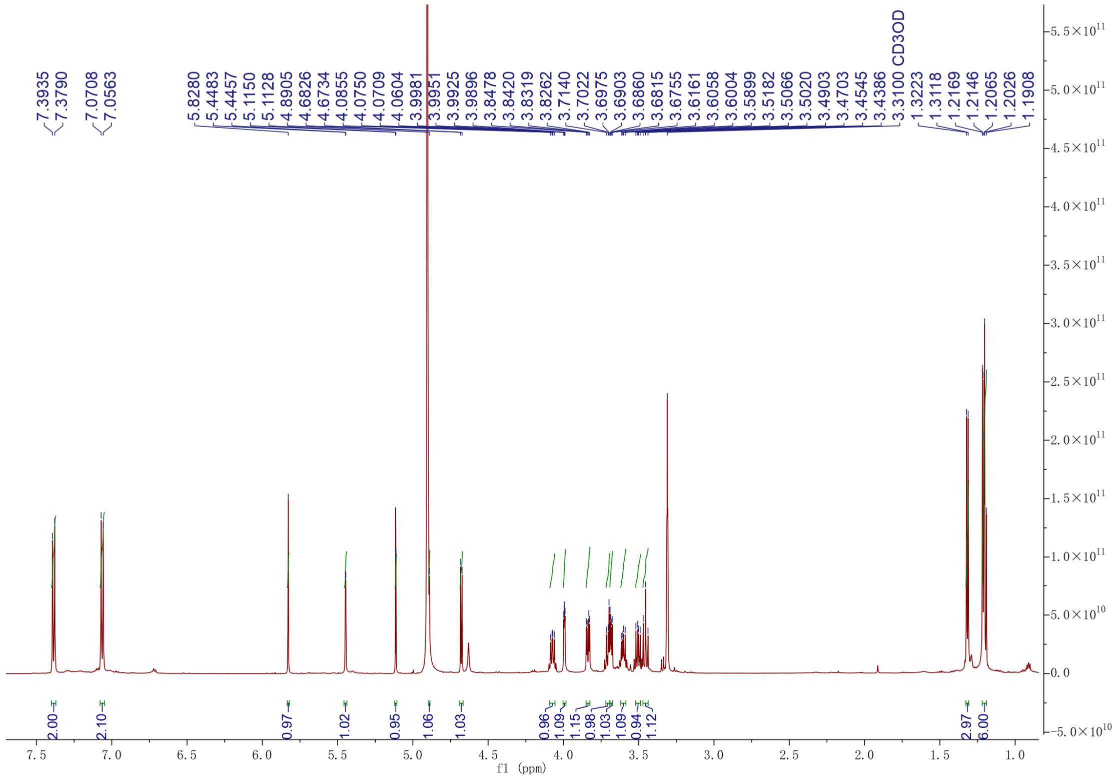


**Figure S12**. $^1$H-NMR (600MHz, $CD_3OD$) spectrum of compound **2**.

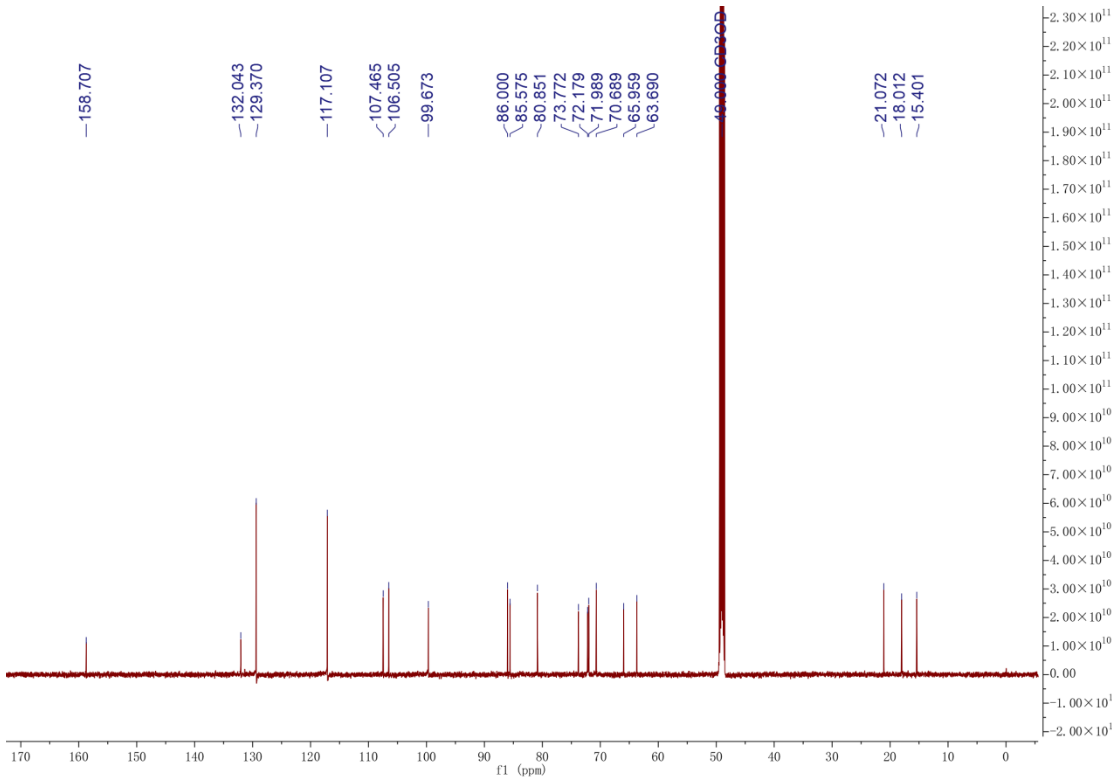


**Figure S13**. $^{13}$C-NMR (150MHz, $CD_3OD$) spectrum of compound **2**.

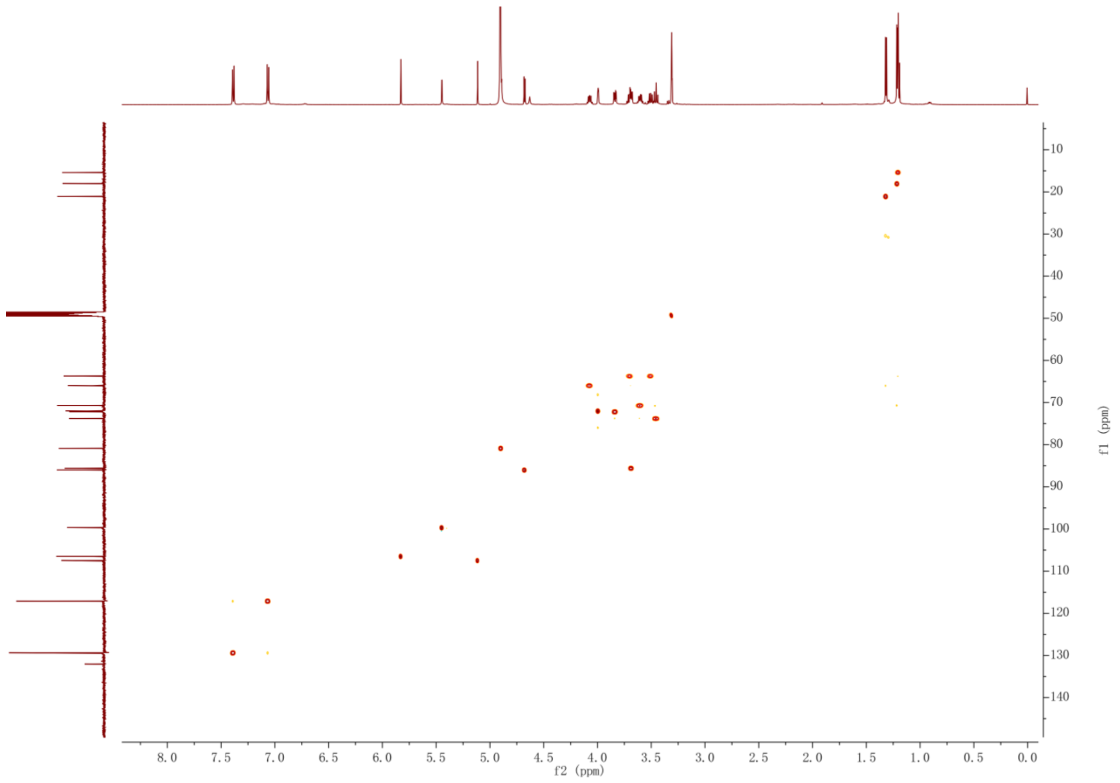


**Figure S14**. HMQC spectrum of compound **2**.

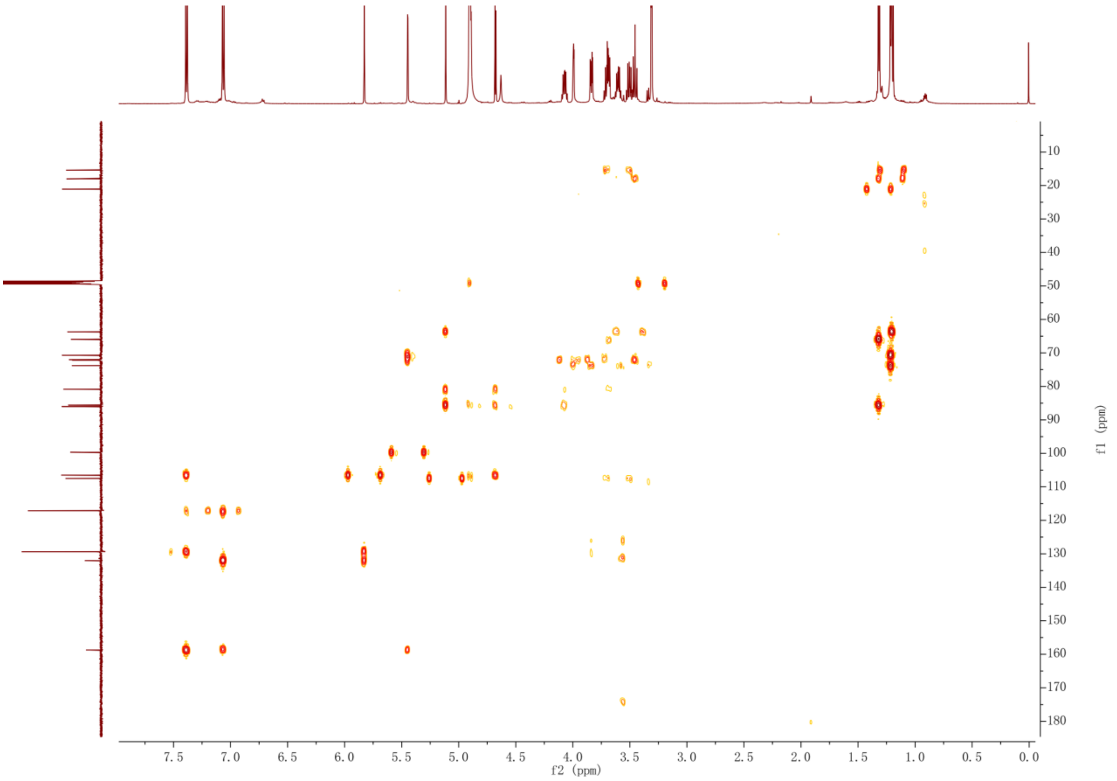


**Figure S15**. HMBC spectrum of compound **2**.

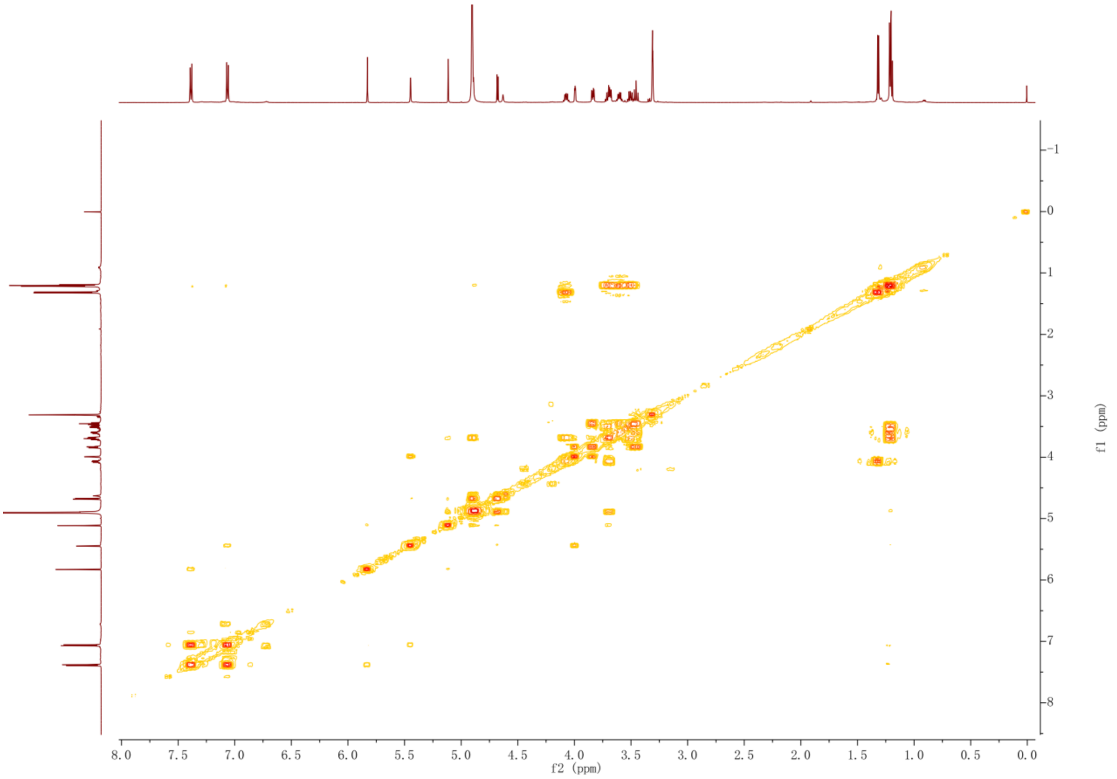


**Figure S16**. $^{1}H$-$^{1}H$ COSY spectrum of compound **2**.

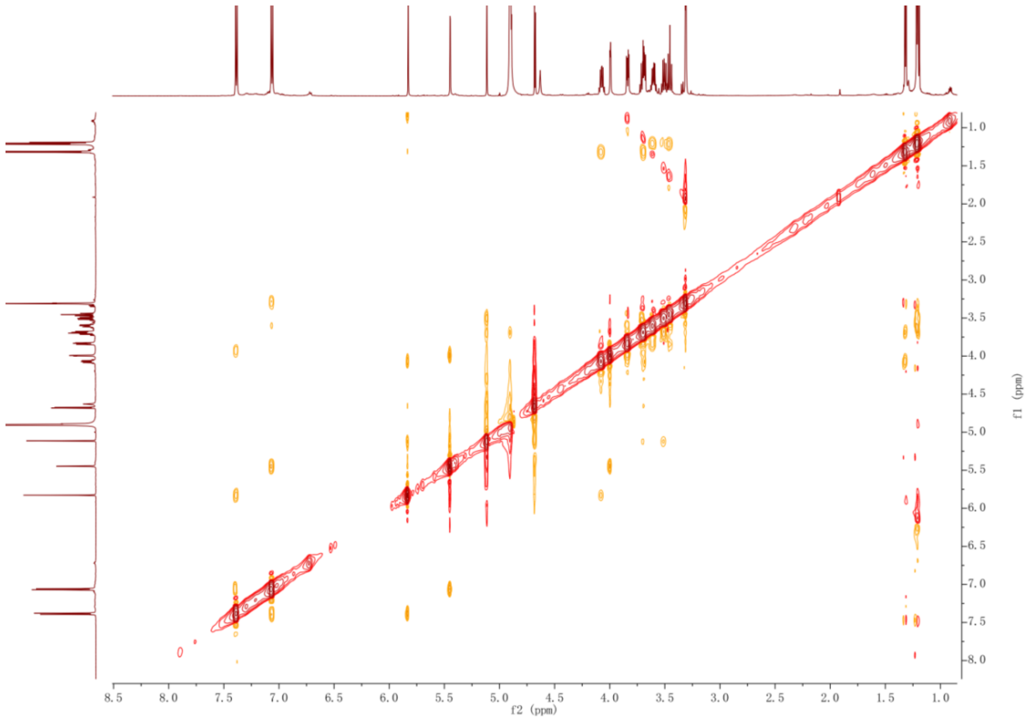


**Figure S17**. NOESY spectrum of compound **2**.

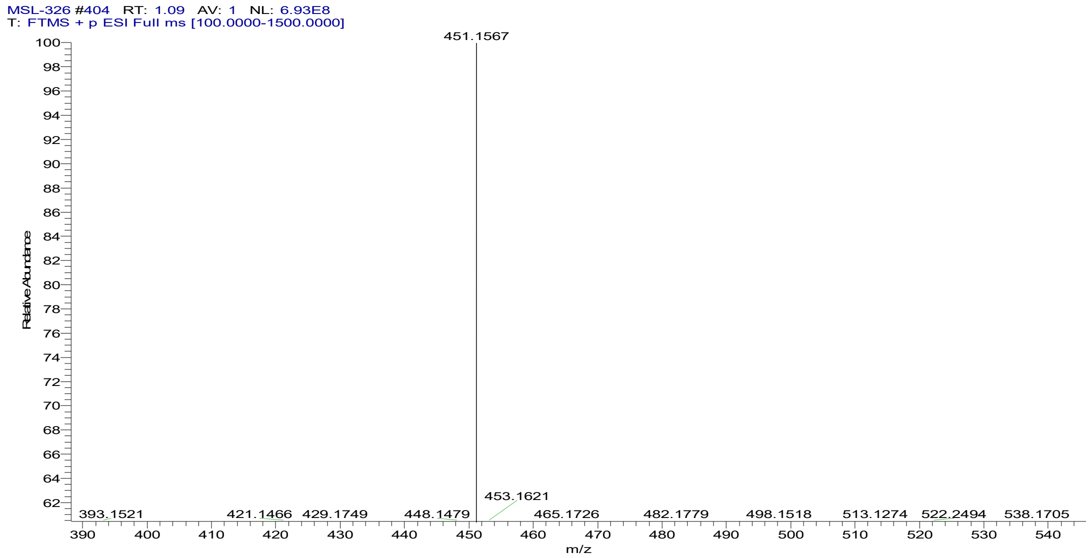


**Figure S18**. HR-ESI-MS spectrum of compound **3** (postive mode).

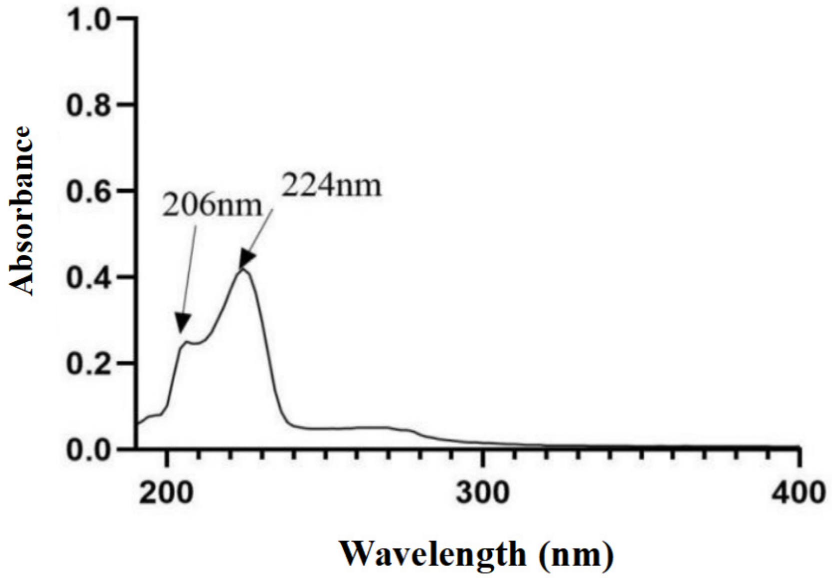


**Figure S19.** UV spectrum of compound **3**.

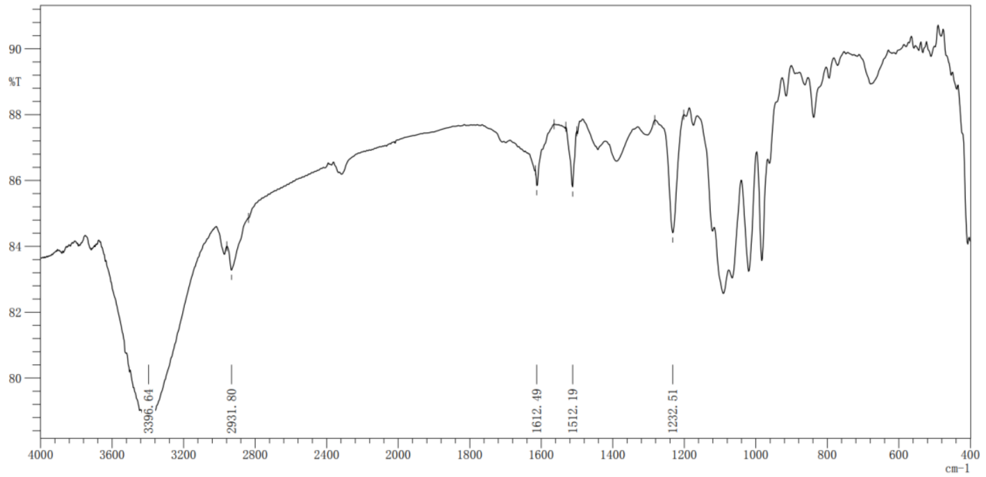


**Figure S20.** IR spectrum of compound **3**.

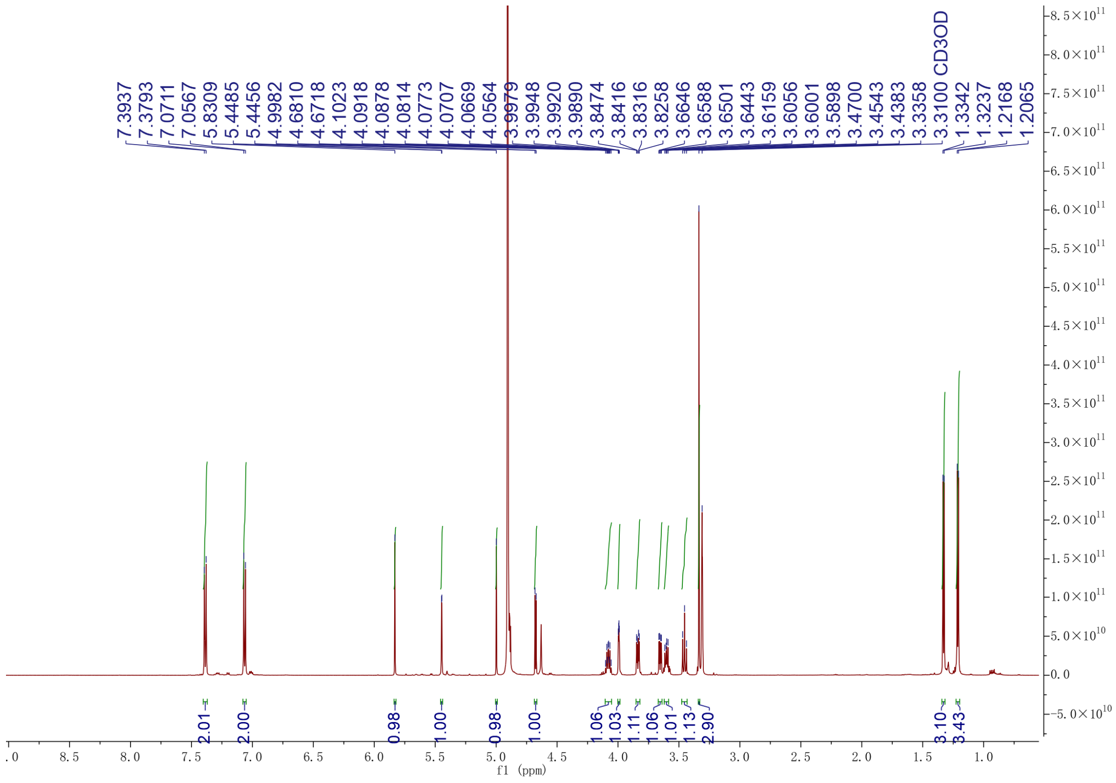

**Figure S21.** $^{1}$H-NMR (600MHz, $CD_3OD$) spectrum of compound **3**.

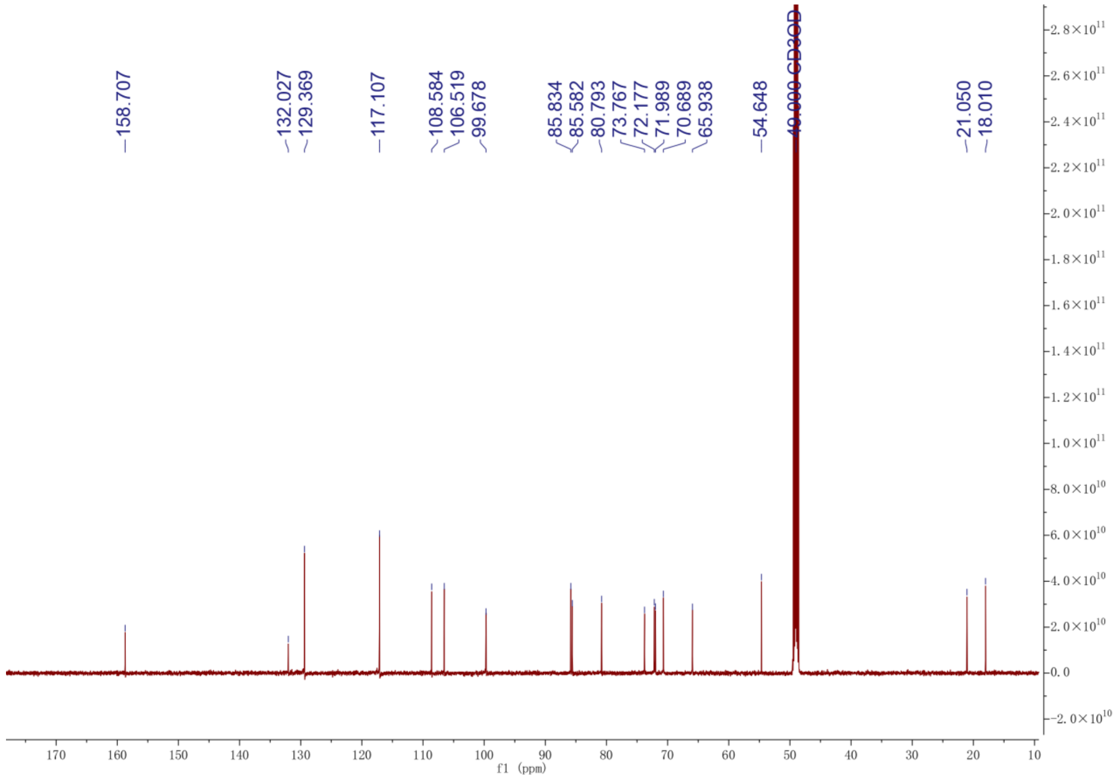

**Figure S22.** $^{13}$C-NMR (150MHz, $CD_3OD$) spectrum of compound **3**.

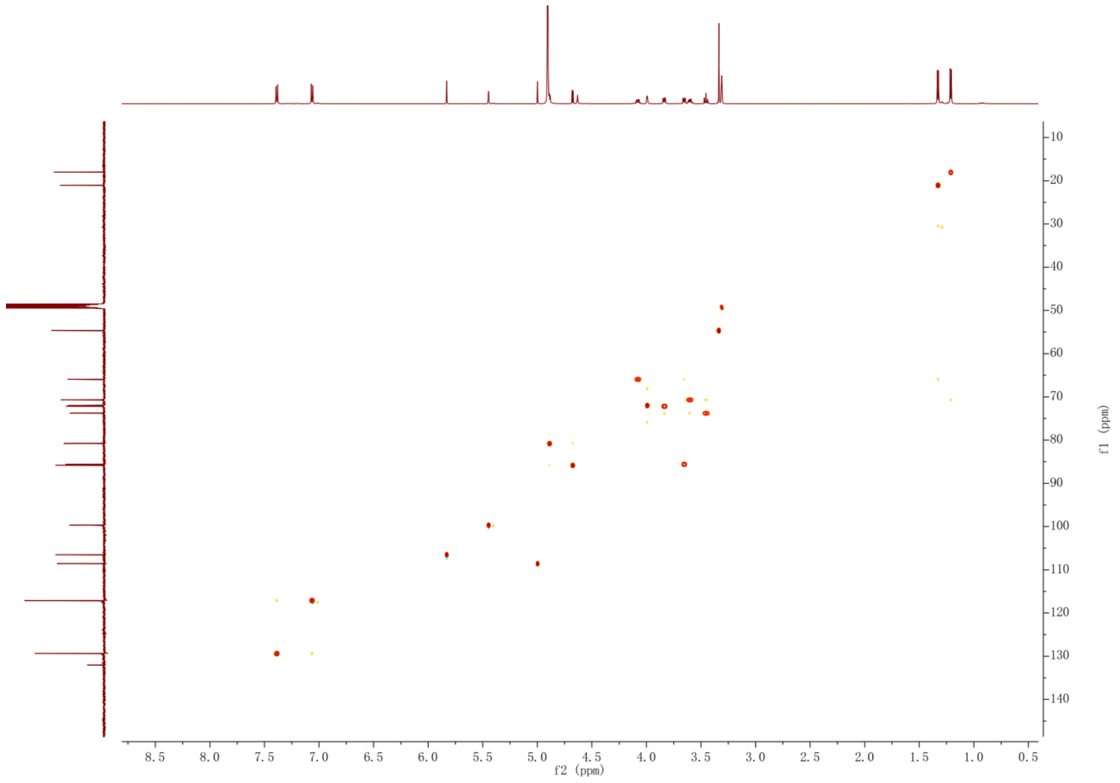


**Figure S23.** HMQC spectrum of compound **3**.

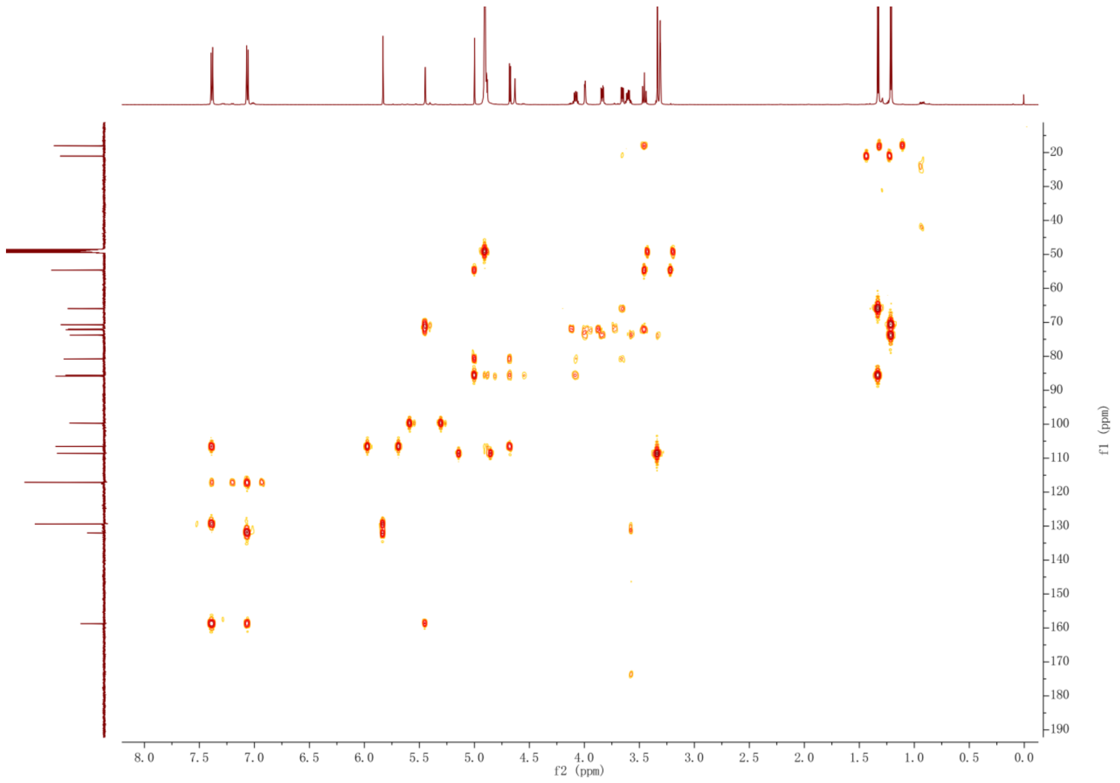


**Figure S24.** HMBC spectrum of compound **3**.

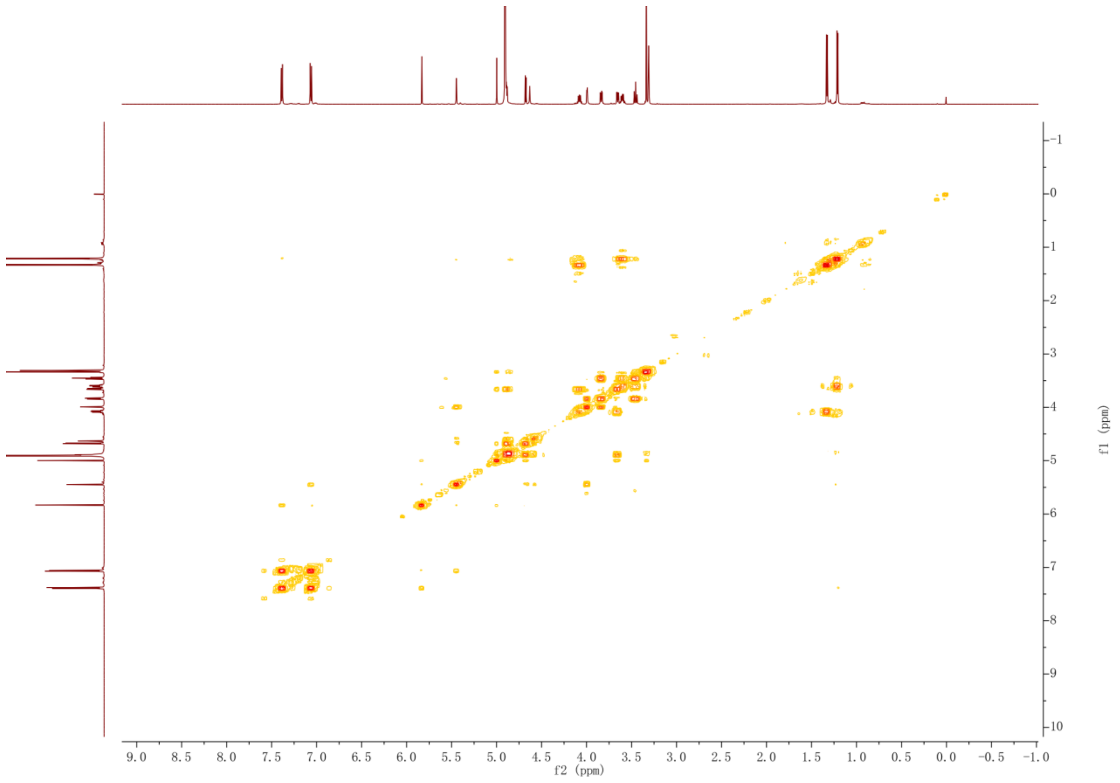


**Figure S25.** $^{1}H$-$^{1}H$ COSY spectrum of compound **3**.

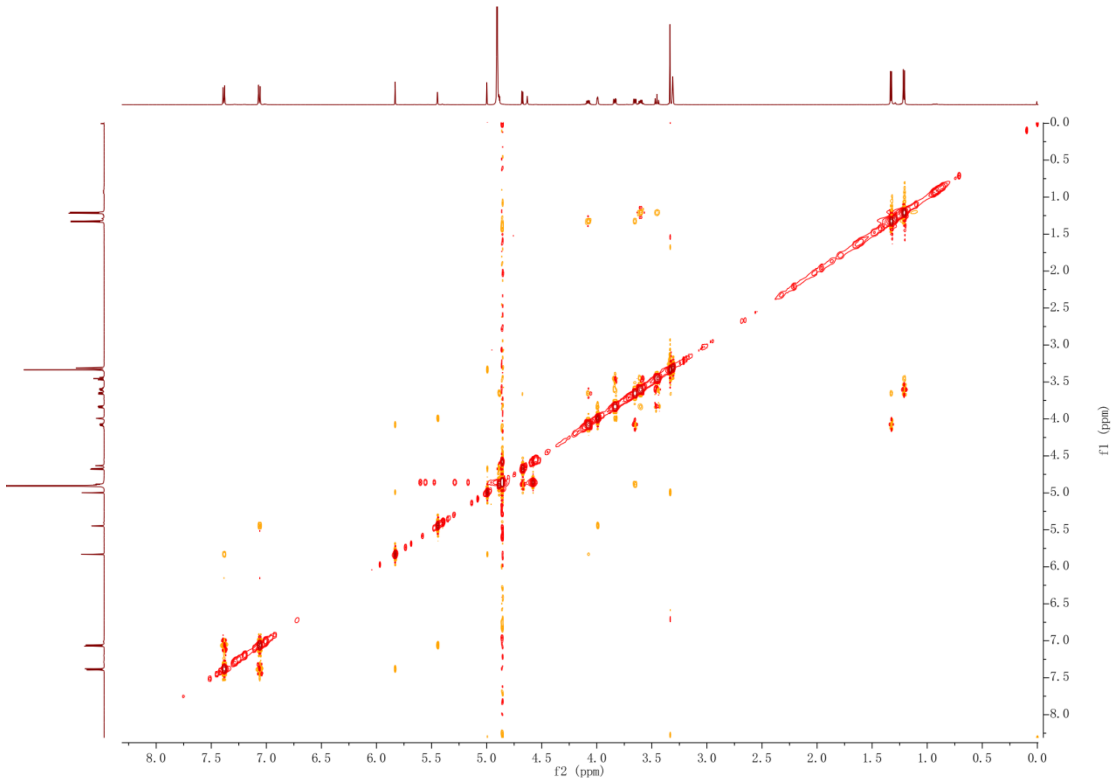


**Figure S26.** NOESY spectrum of compound **3**.

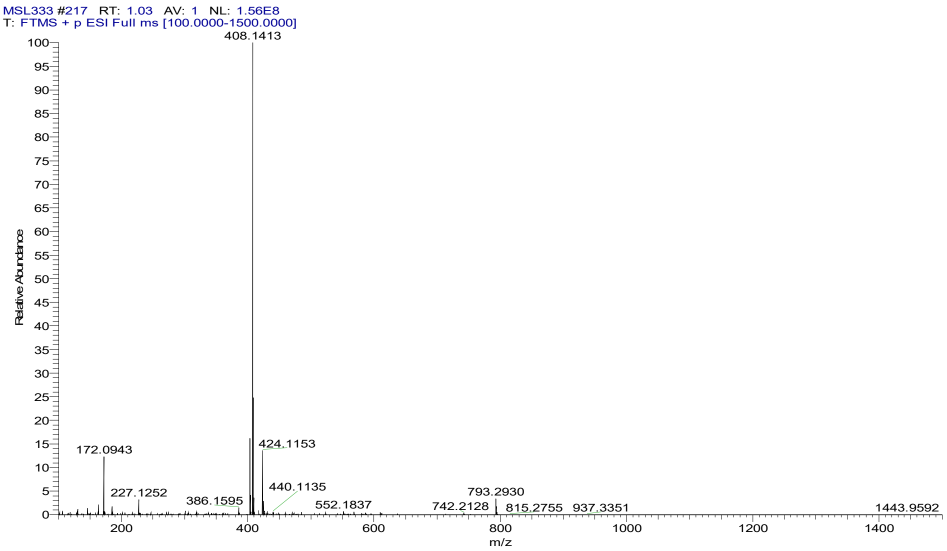


**Figure S27.** HR-ESI-MS spectrum of compound **4** (postive mode).

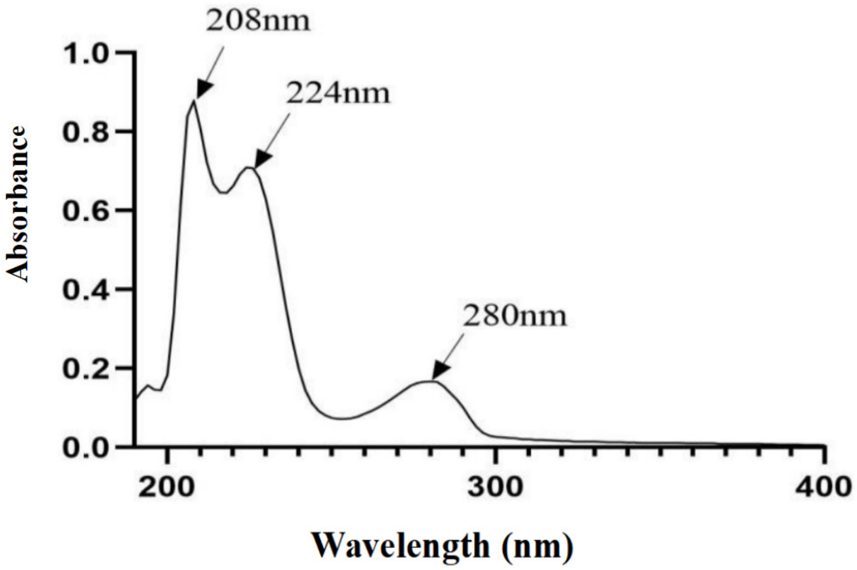


**Figure S28.** UV spectrum of compound **4**.

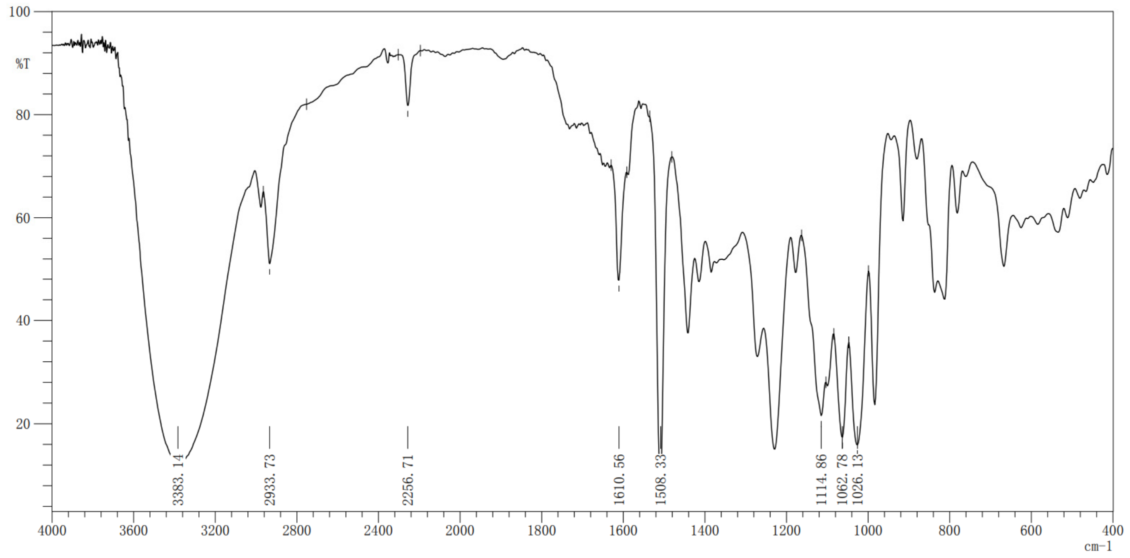


**Figure S29.** IR spectrum of compound **4**.

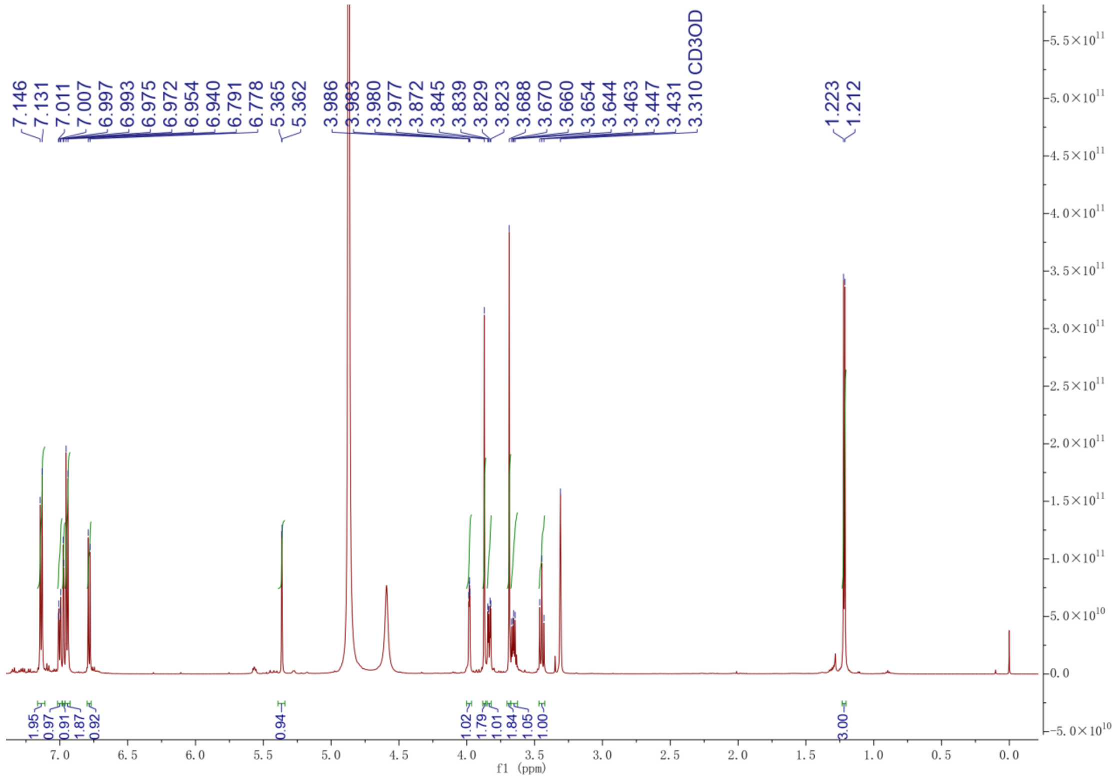


**Figure S30.** $^{1}$H-NMR (600MHz, $CD_3OD$) spectrum of compound **4**.

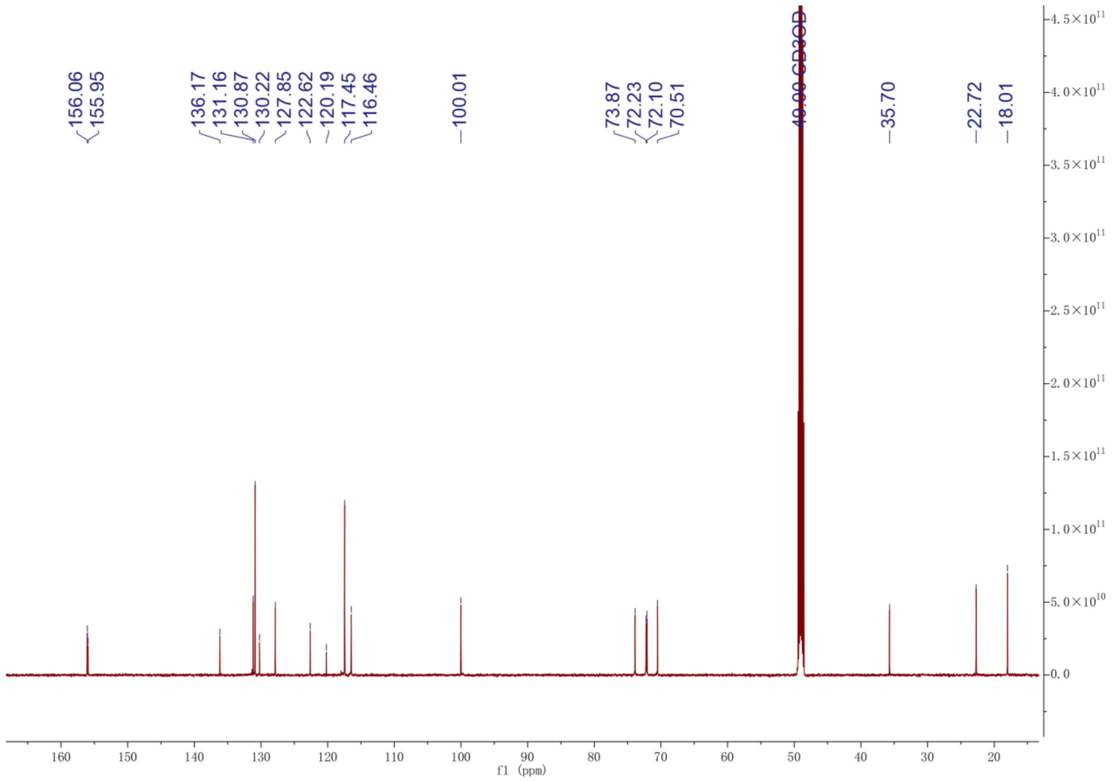


**Figure S31.** $^{13}$C-NMR (150MHz, $CD_3OD$) spectrum of compound **4**.

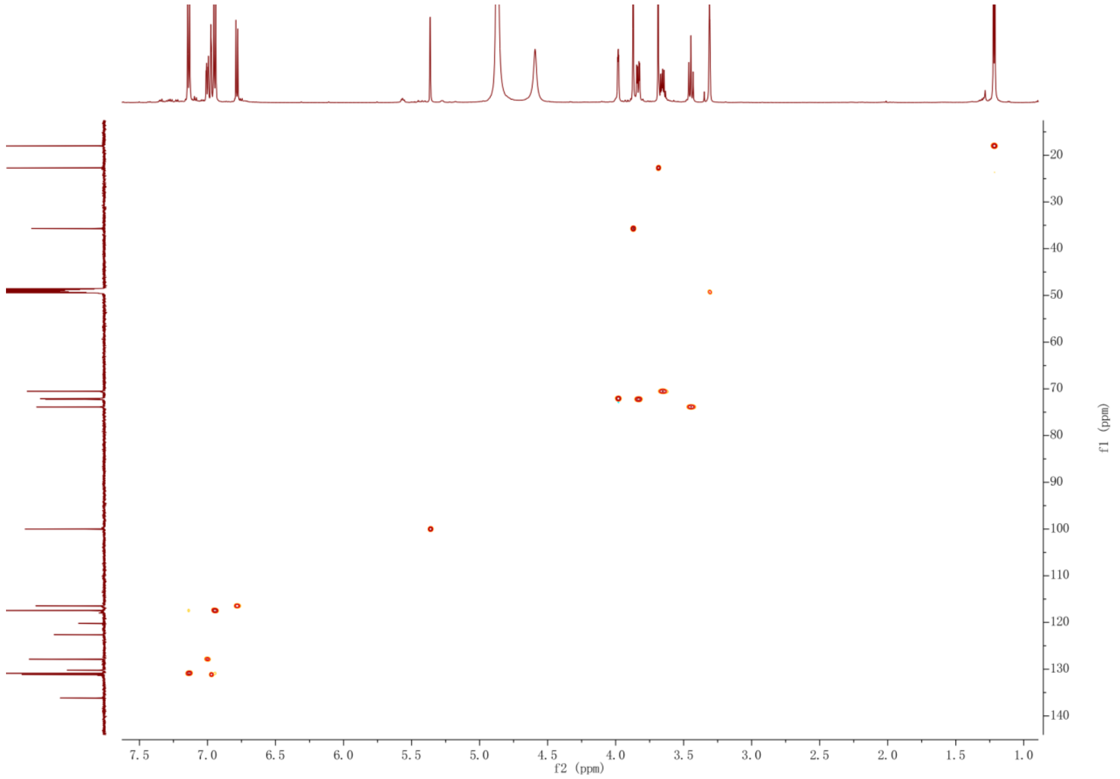


**Figure S32.** HMQC spectrum of compound **4**.

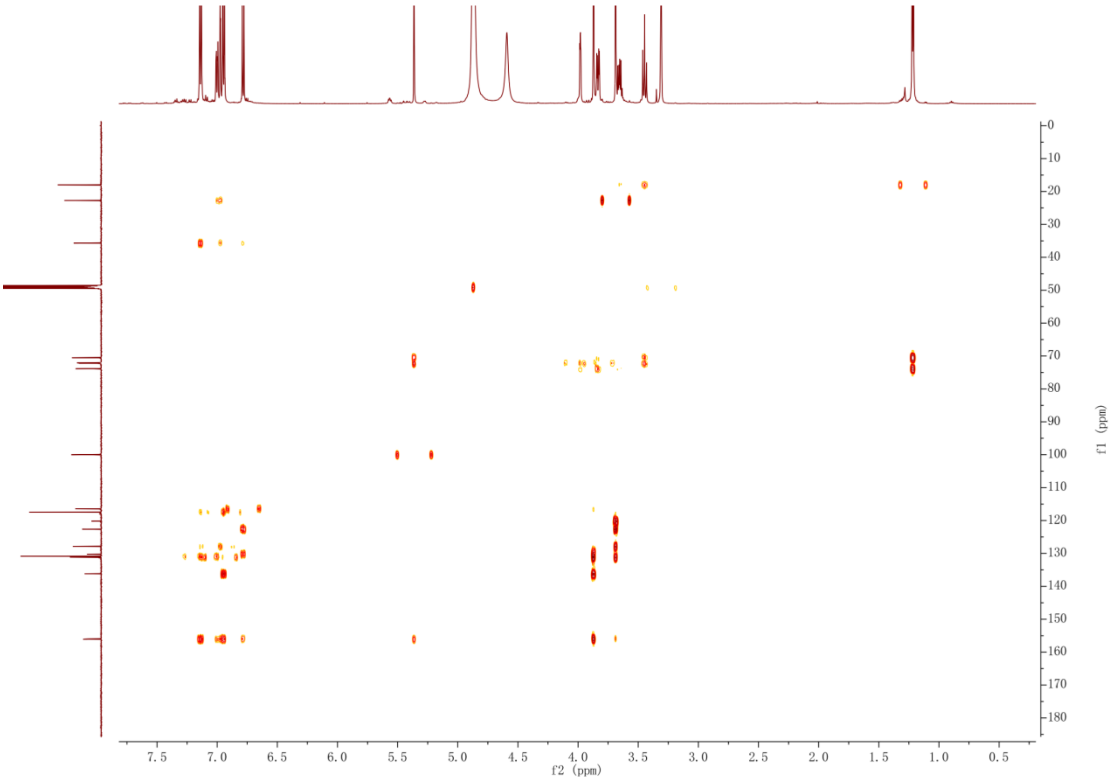


**Figure S33.** HMBC spectrum of compound **4**.

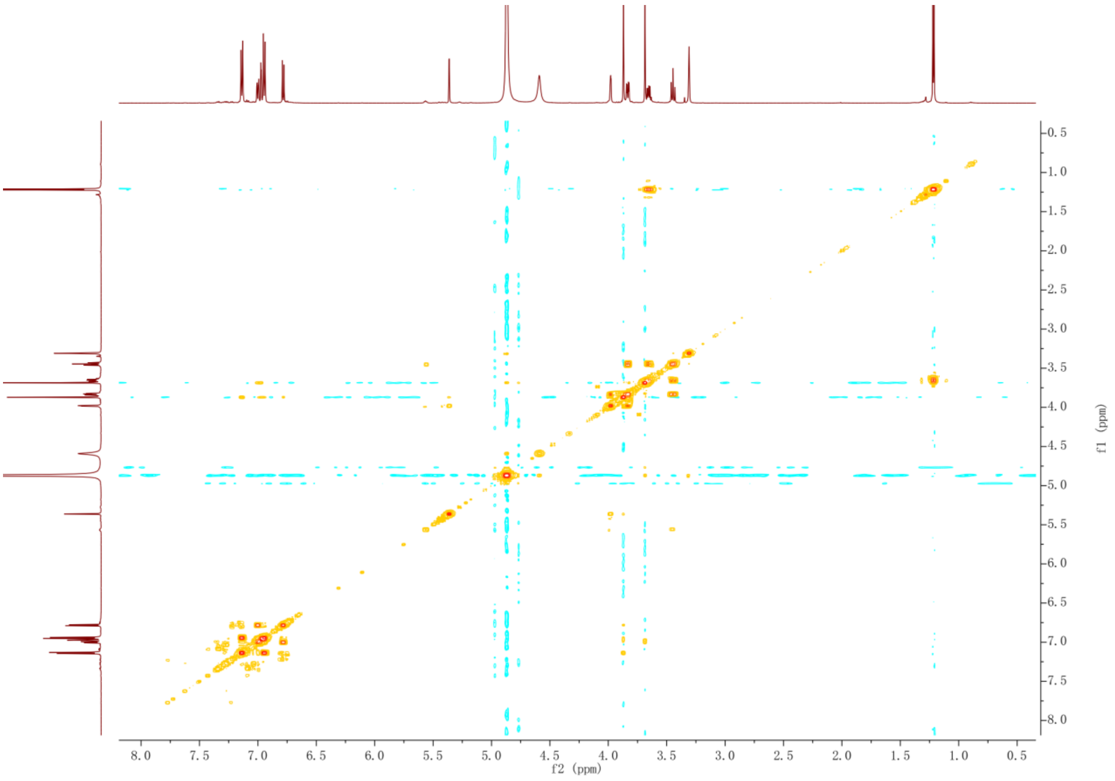


**Figure S34.** $^{1}$H-$^{1}$H COSY spectrum of compound **4**.

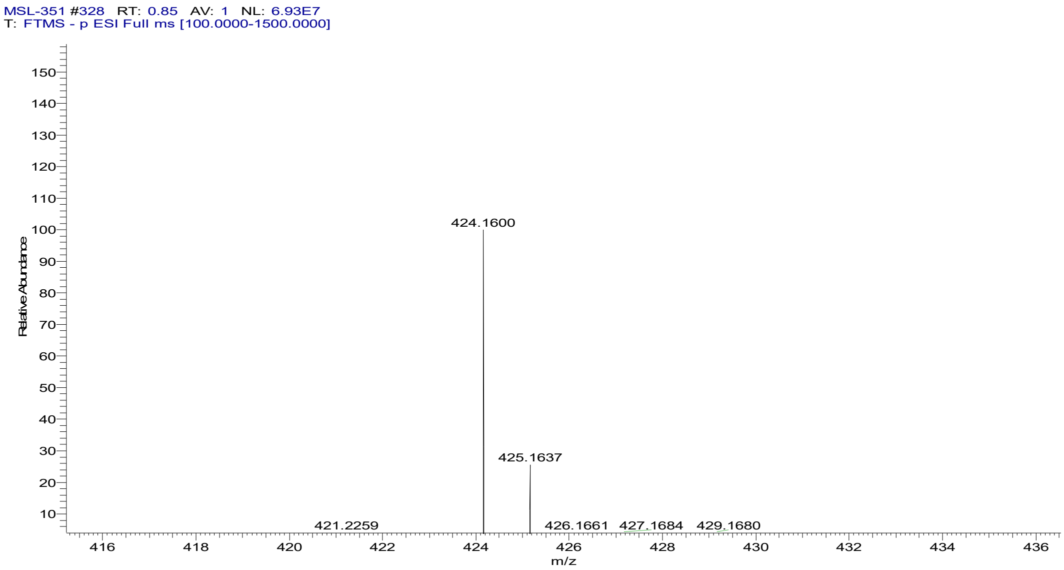


**Figure S35.** HR-ESI-MS spectrum of compound **5** (negative mode).

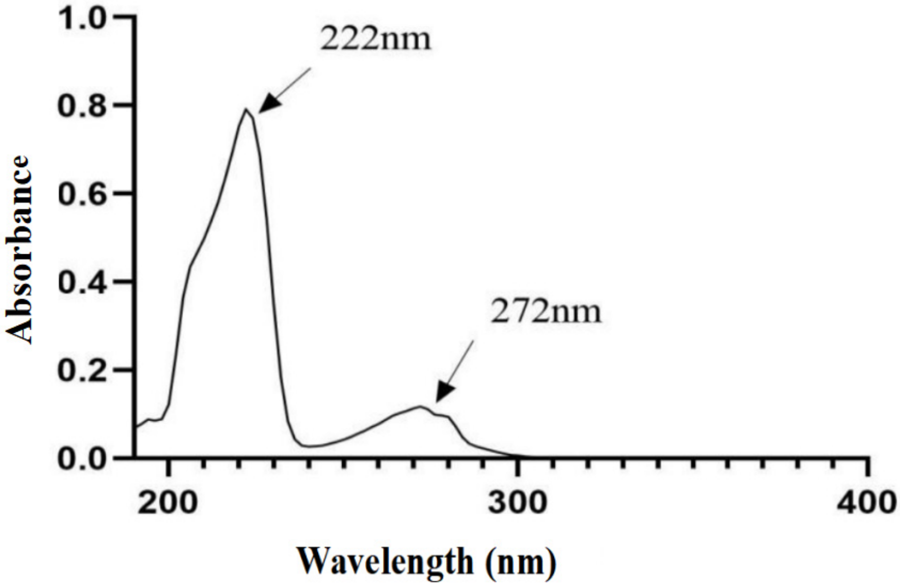


**Figure S36.** UV spectrum of compound **5**.

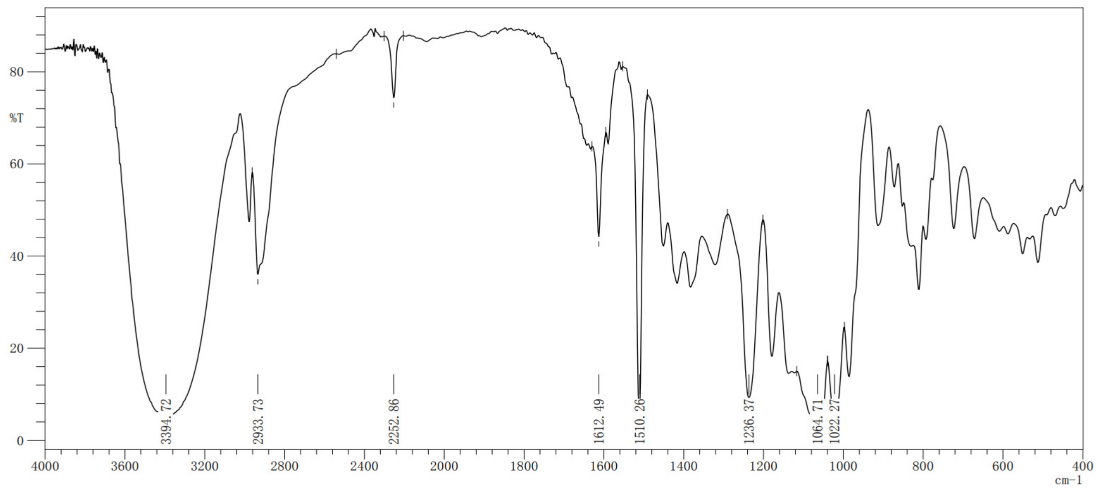


**Figure S37.** IR spectrum of compound **5**.

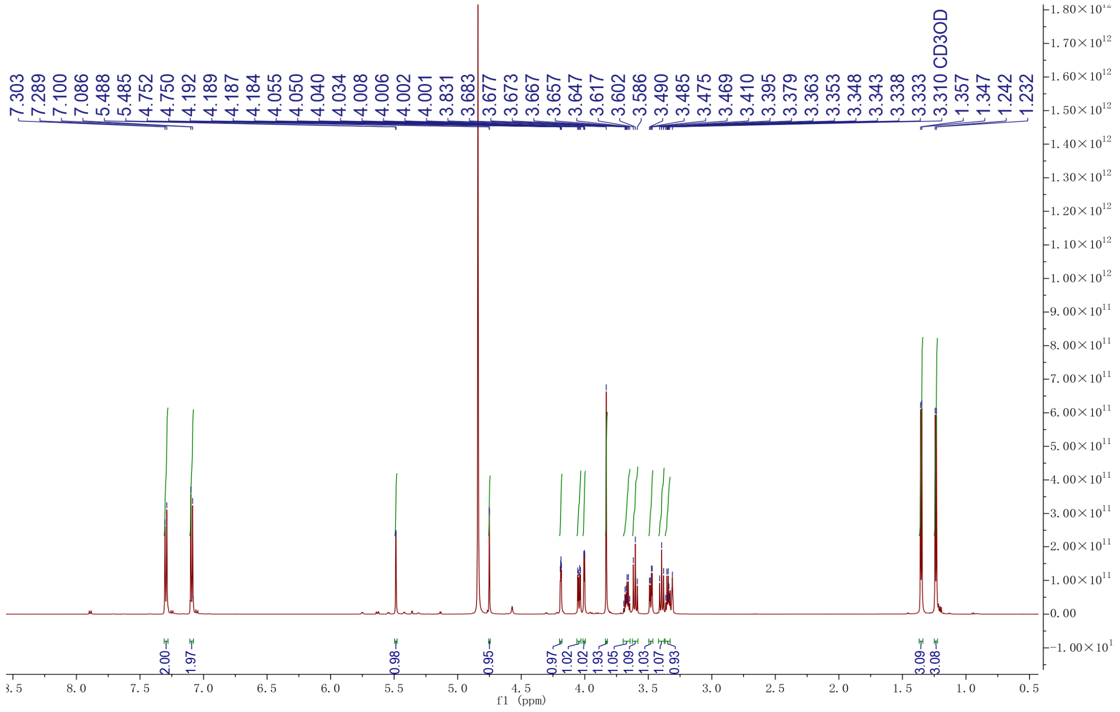


**Figure S38.** $^{1}$H-NMR (600MHz, $CD_3OD$) spectrum of compound **5**.

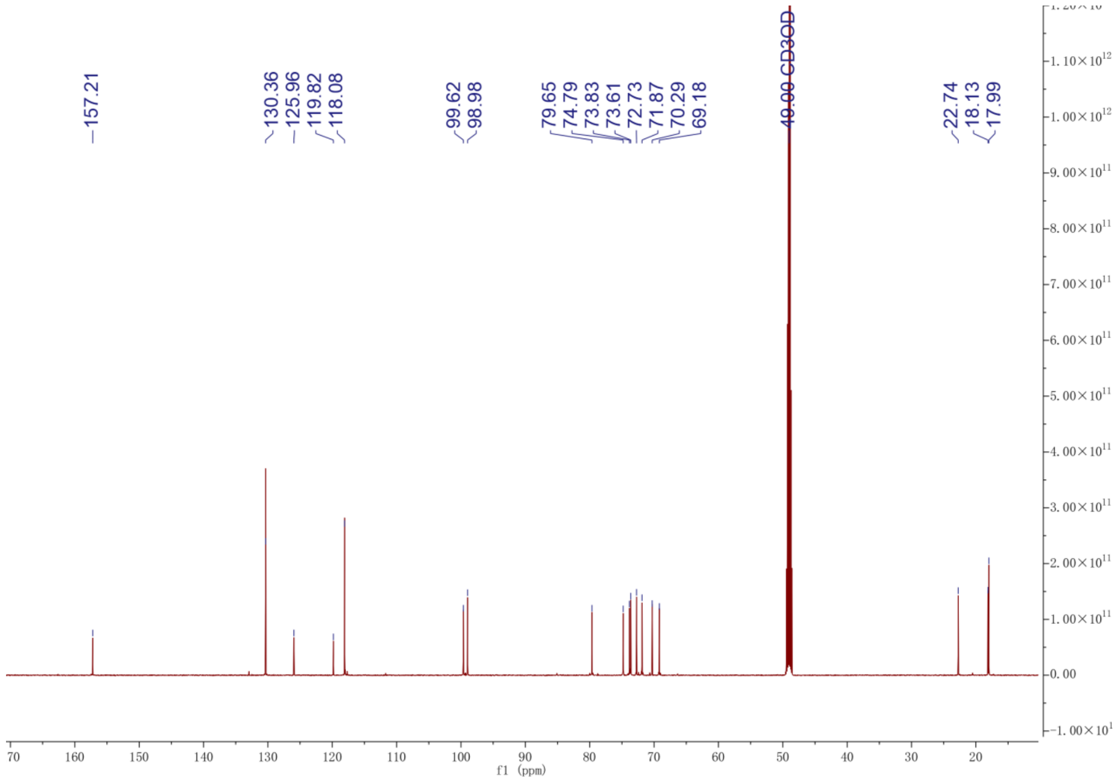


**Figure S39.** $^{13}$C-NMR (150MHz, $CD_3OD$) spectrum of compound **5**.

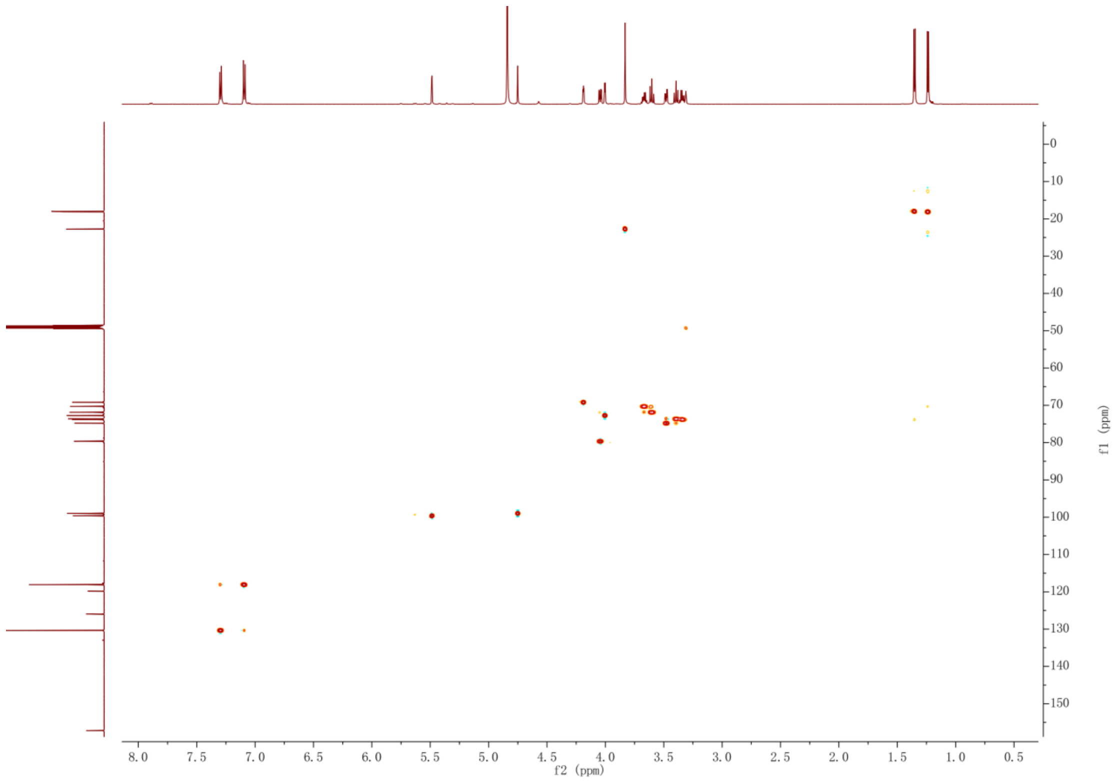


**Figure S40.** HMQC spectrum of compound **5**.

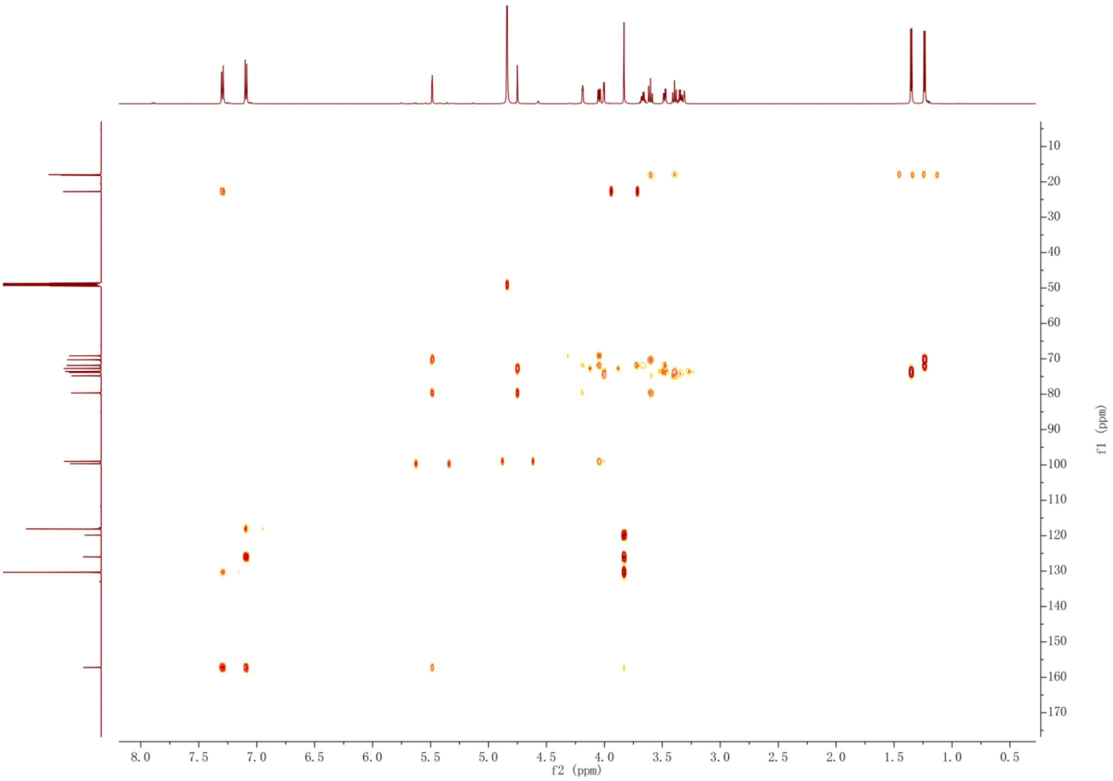


**Figure S41.** HMBC spectrum of compound **5**.

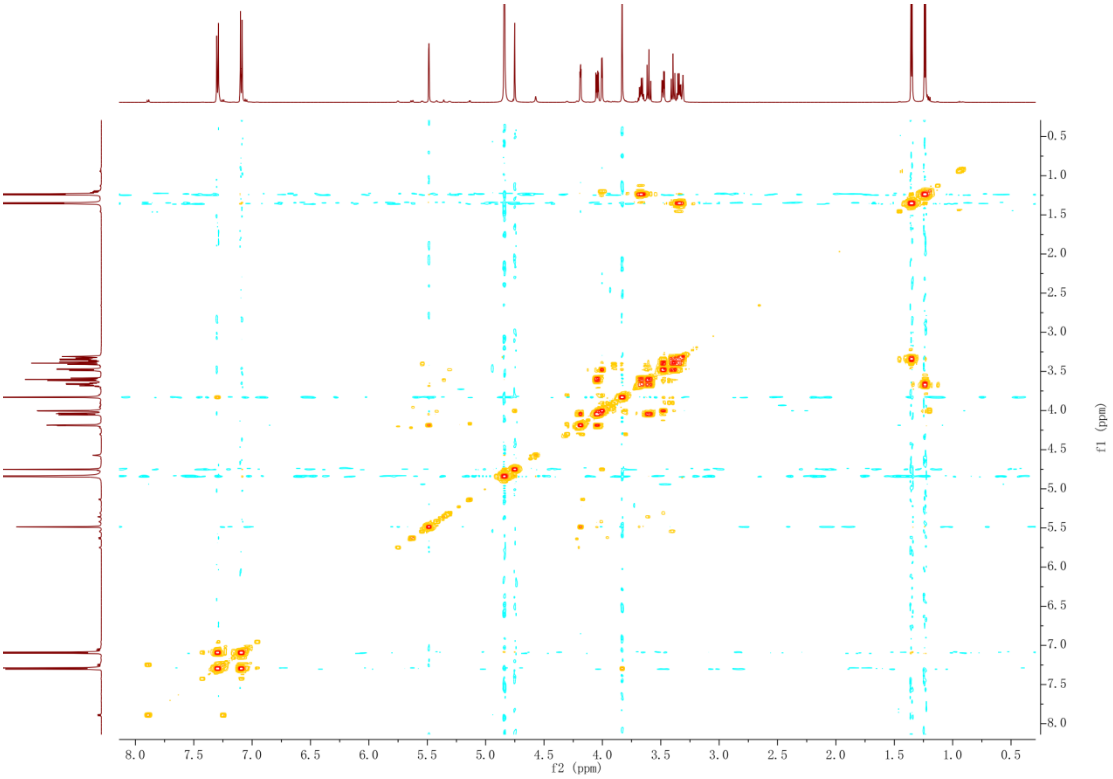


**Figure S42.** ¹H-¹H COSY spectrum of compound **5**.

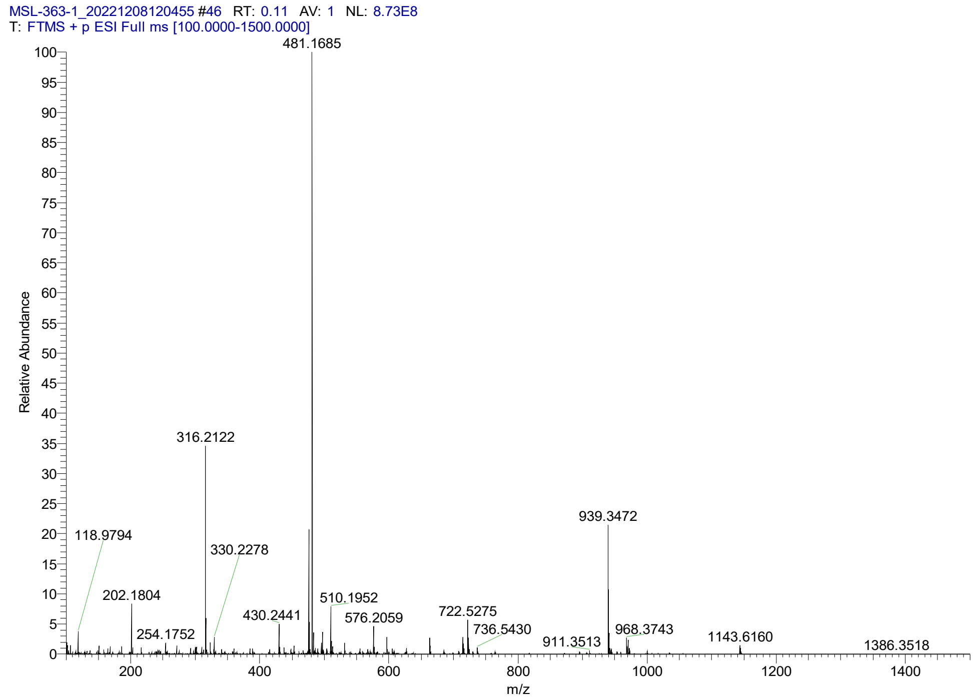


**Figure S43.** HR-ESI-MS spectrum of compound **6** (postive mode).

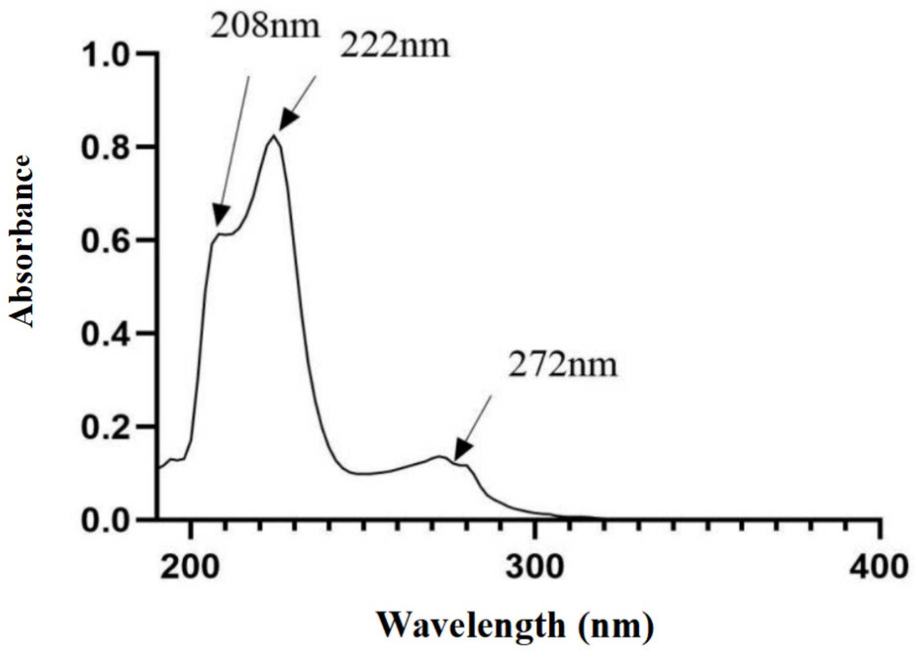


**Figure S44.** UV spectrum of compound **6**.

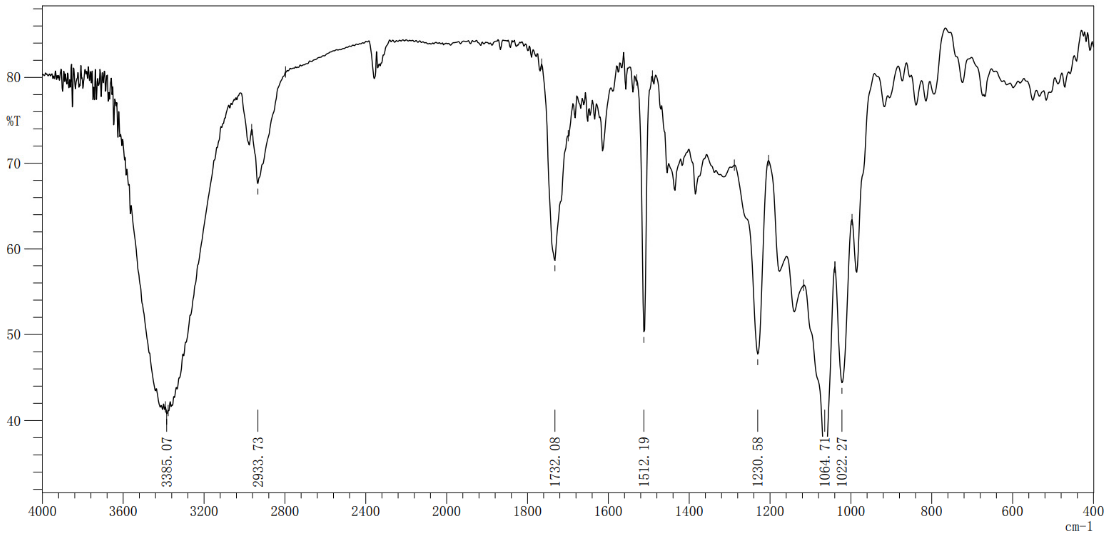


**Figure S45.** IR spectrum of compound **6**.

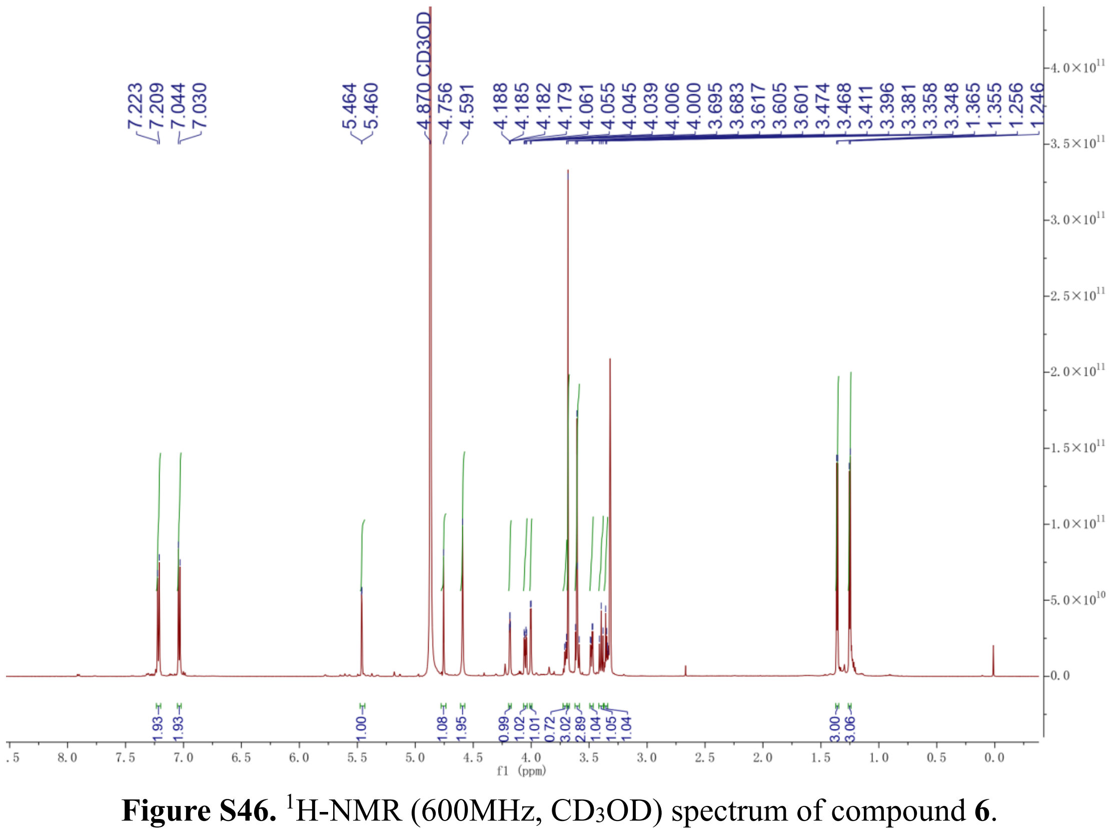


**Figure S46.** $^{1}$H-NMR (600MHz, $CD_3OD$) spectrum of compound **6**.

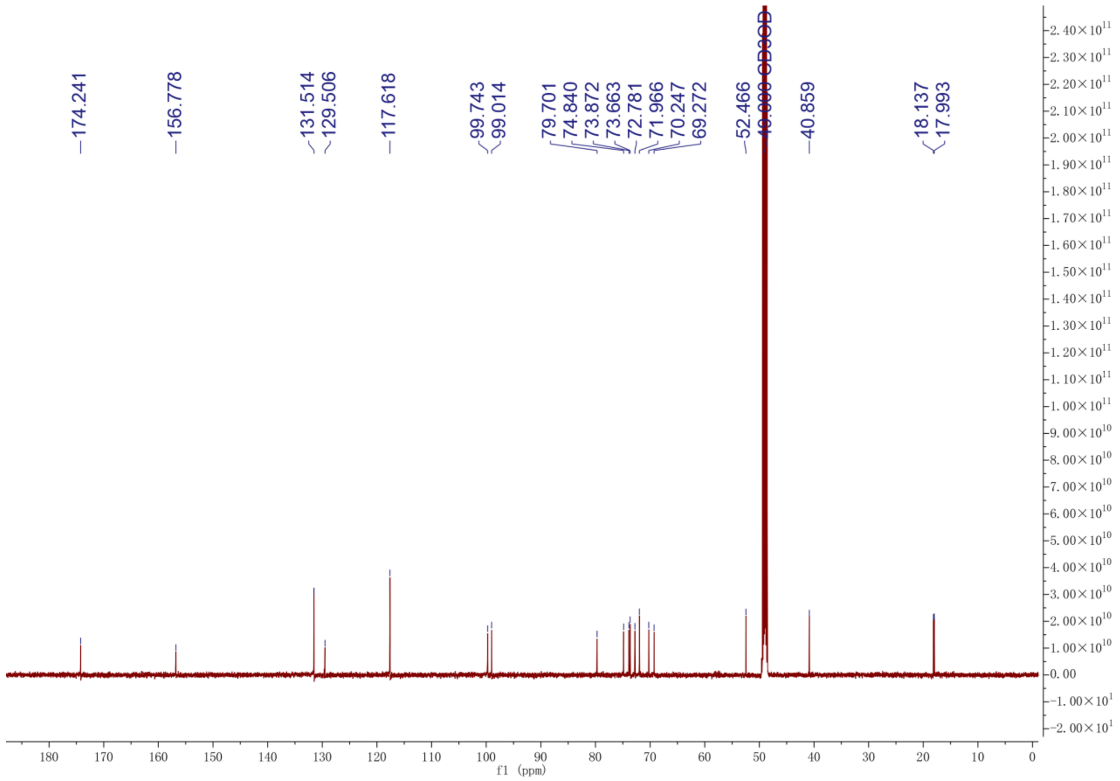


**Figure S47.** $^{13}$C-NMR (150MHz, CD3OD) spectrum of compound **6**.

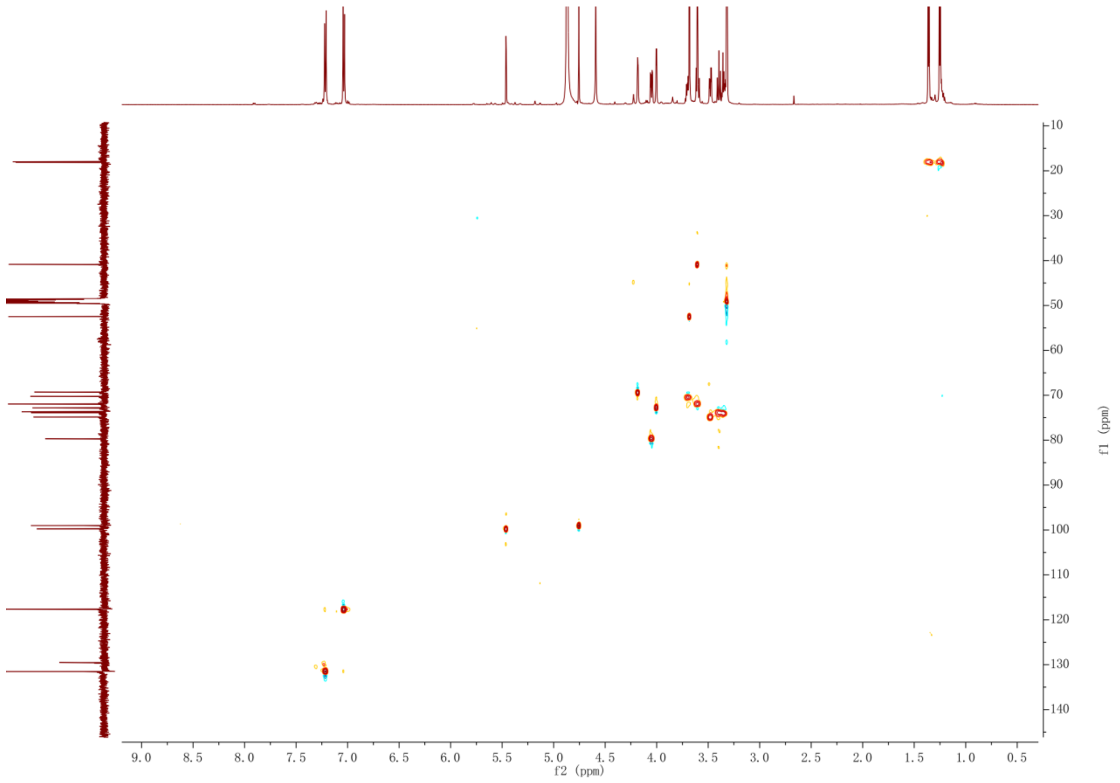


**Figure S48.** HMQC spectrum of compound **6**.

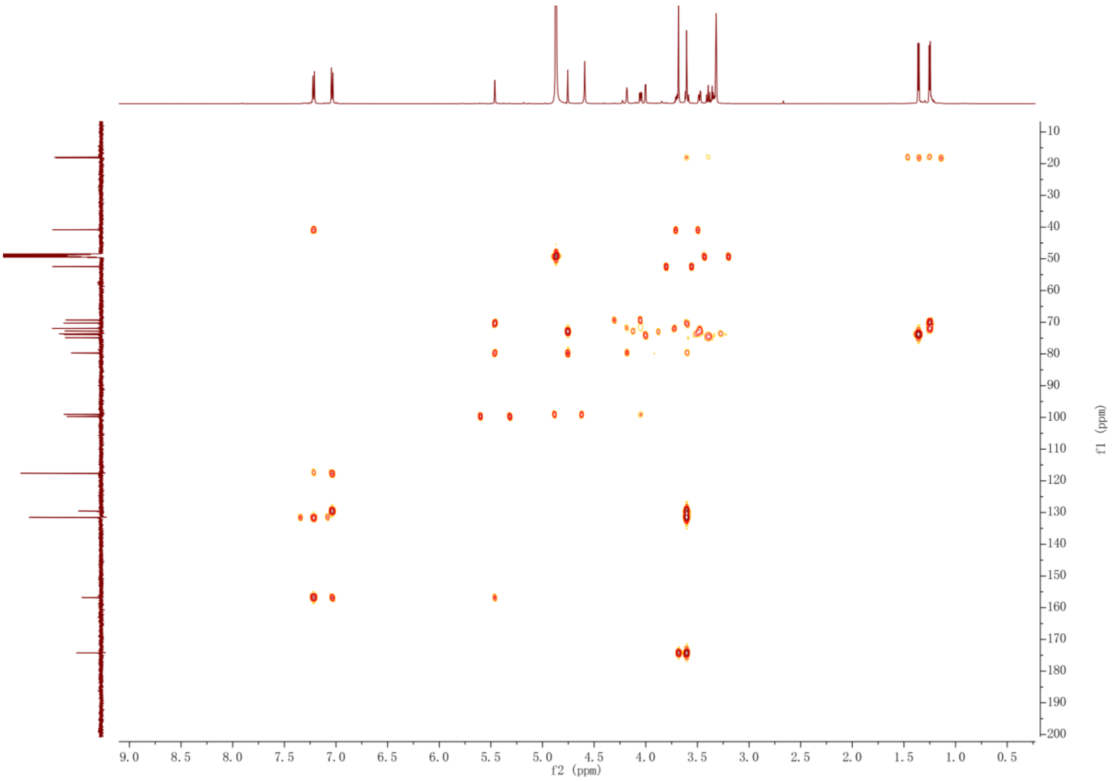


**Figure S49.** HMBC spectrum of compound **6**.

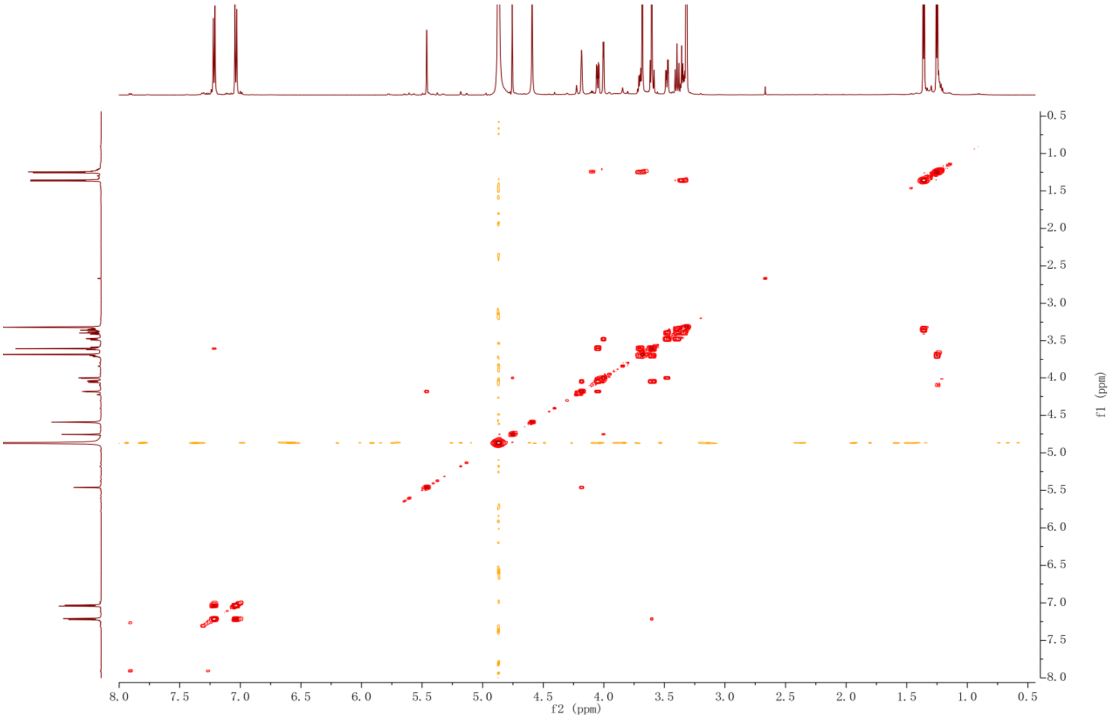


**Figure S50.** $^1$H-$^1$H COSY spectrum of compound **6**.

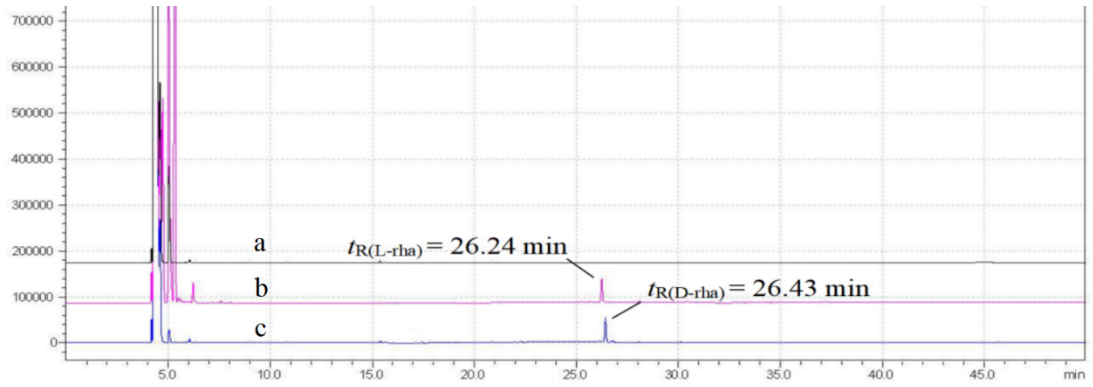


D-rhamnose and L-rhamnose (contrast with compound **1-3**)
(a: blank; b: L-rhamnose; c: D-rhamnose)

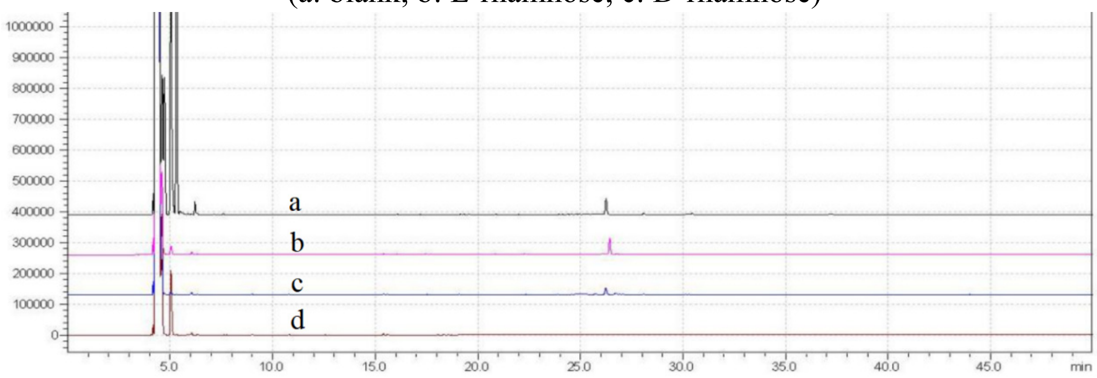


compound **1**
(a: L-rhamnose; b: D-rhamnose; c: compound **1**; d: blank)

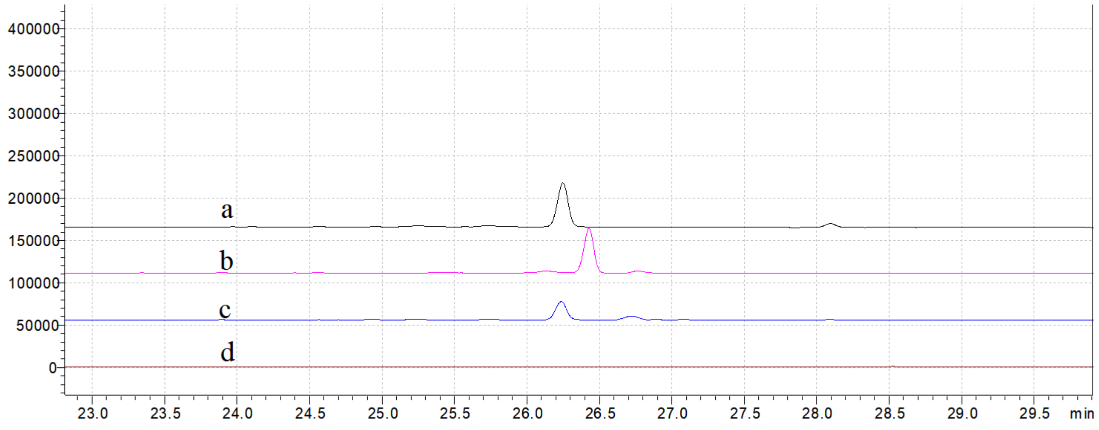


Extended compound **1**
(a: L-rhamnose; b: D-rhamnose; c: compound **1**; d: blank)

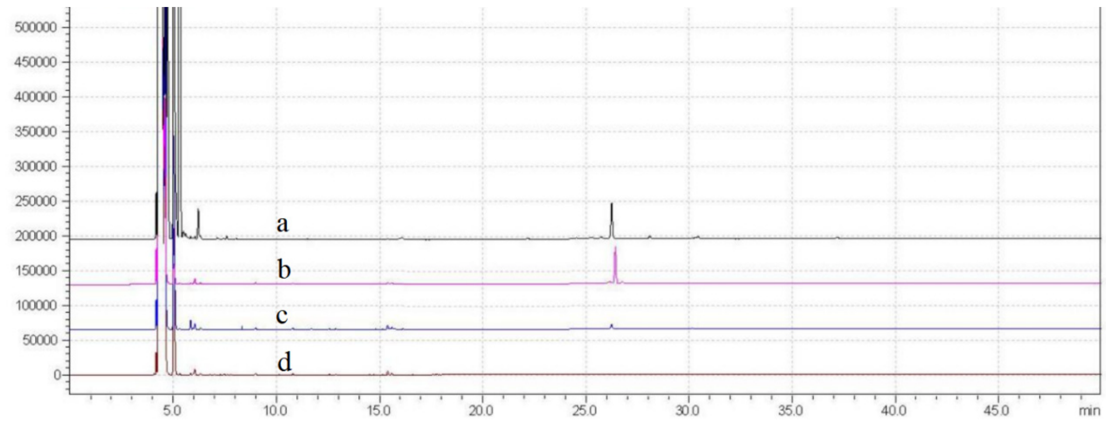


compound **2**
(a: L-rhamnose; b: D-rhamnose; c: compound **2**; d: blank)

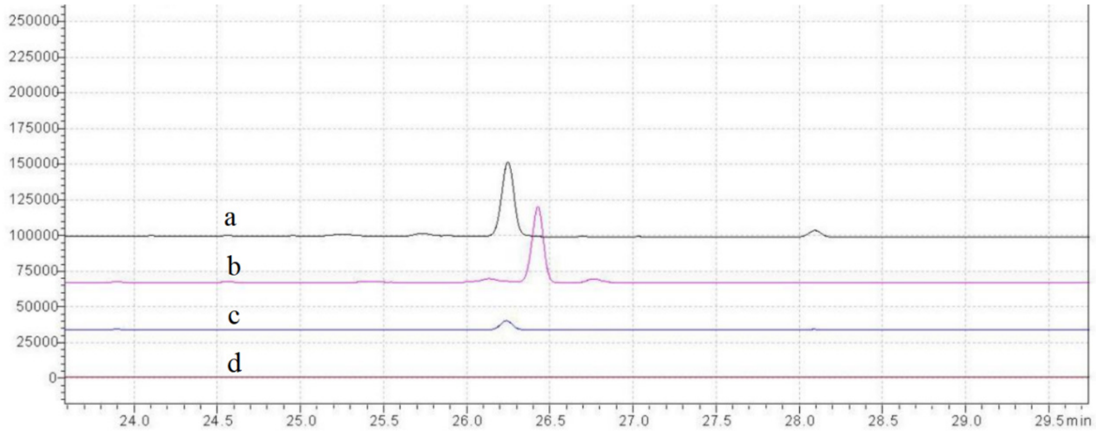


Extended compound **2**
(a: L-rhamnose; b: D-rhamnose; c: compound **2**; d: blank)

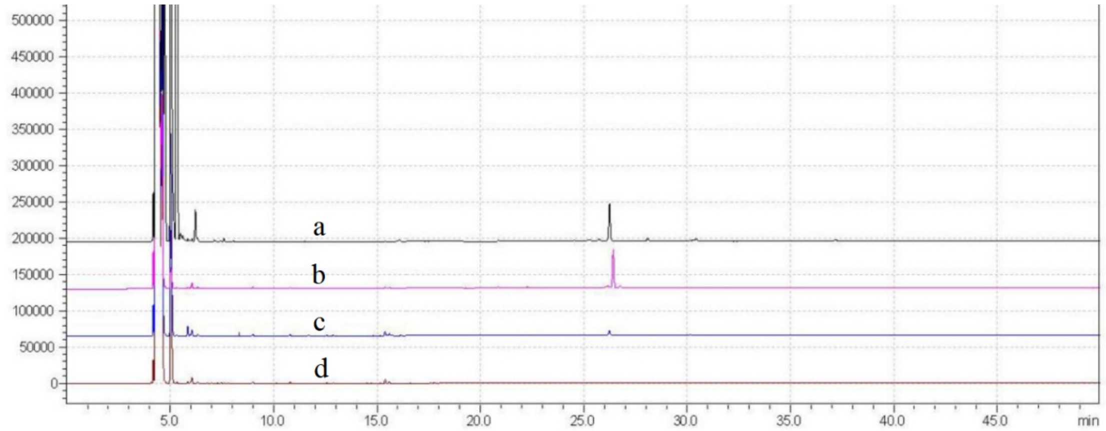


compound **3**
(a: L-rhamnose; b: D-rhamnose; c: compound **3**; d: blank)

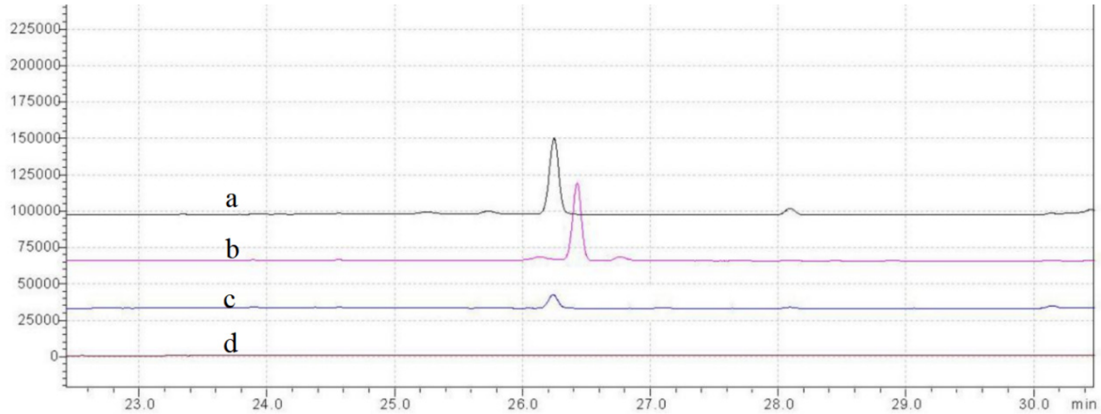


Extended compound **3**

(a: L-rhamnose; b: D-rhamnose; c: compound **3**; d: blank)

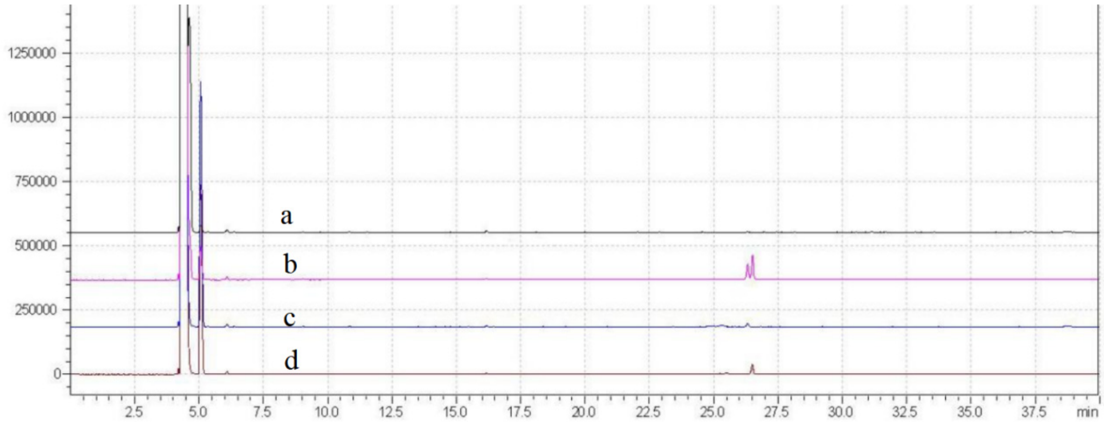


D-rhamnose and L-rhamnose (contrast with compound **4-6**)

(a: blank; b: D-L-rhamnose; c: L-rhamnose; d: D-rhamnose)

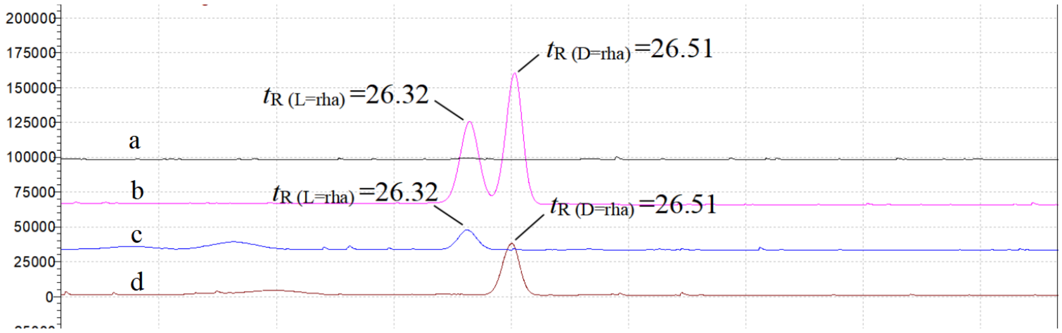


Extended D-rhamnose and L-rhamnose (contrast with compound **4-6**)

(a: blank; b: D-L-rhamnose; c: L-rhamnose; d: D-rhamnose)

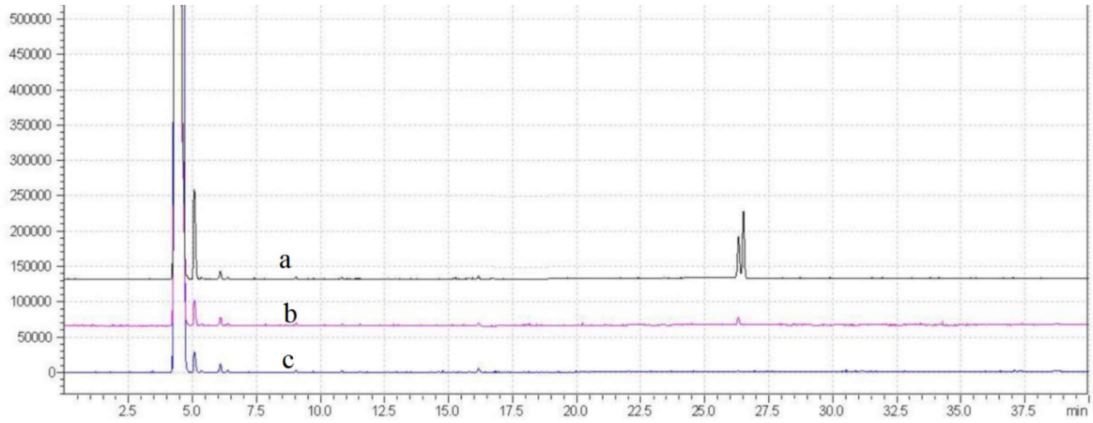


compound **4**
(a: D-L-rhamnose; b: compound **4**; c: blank)

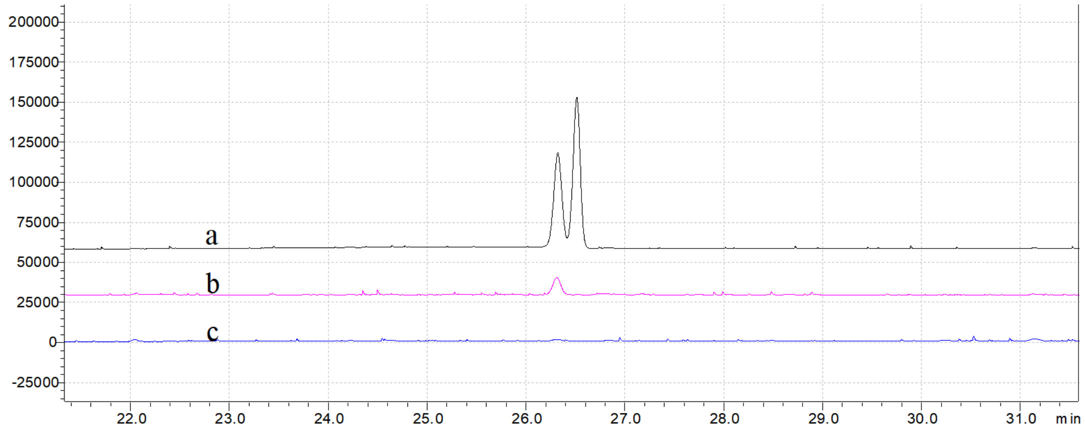


Extended compound **4**
(a: D-L-rhamnose; b: compound **4**; c: blank)

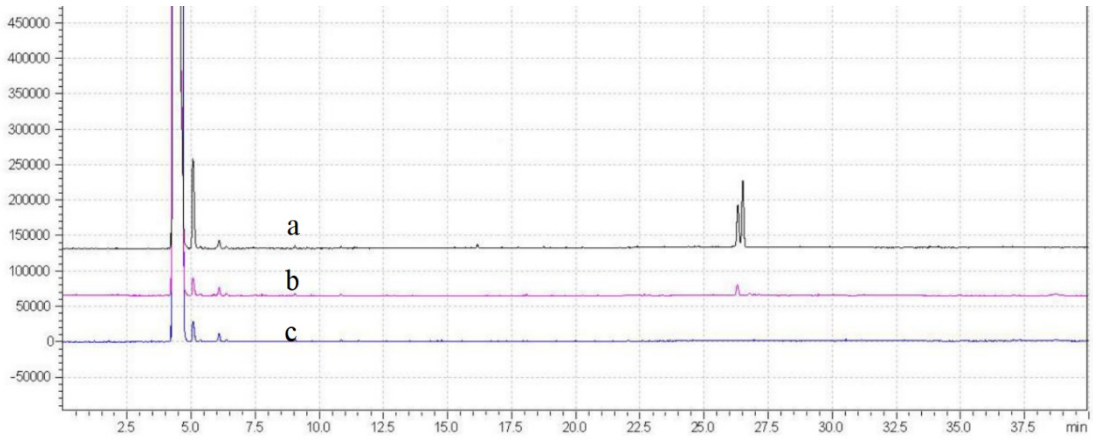


compound **5**
(a: D-L-rhamnose; b: compound **5**; c: blank)

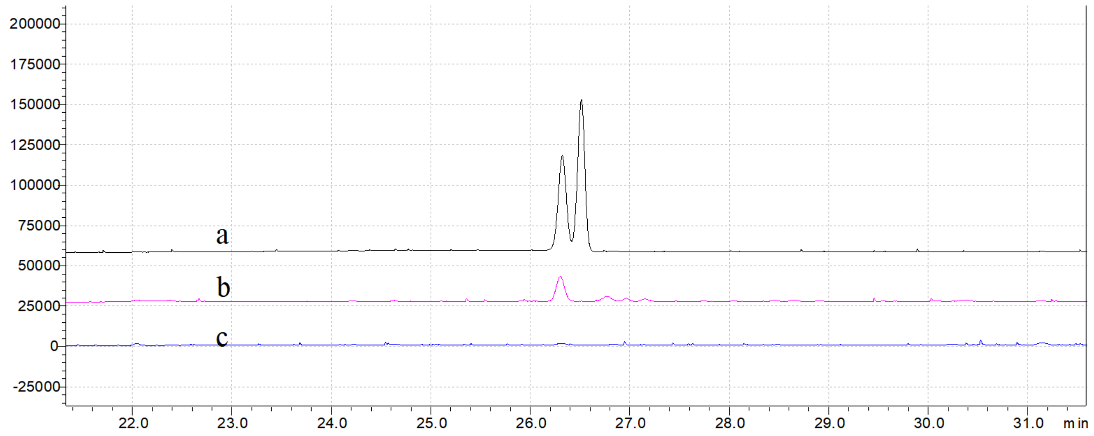


Extended compound **5**
(a: D-L-rhamnose; b: compound **5**; c: blank)

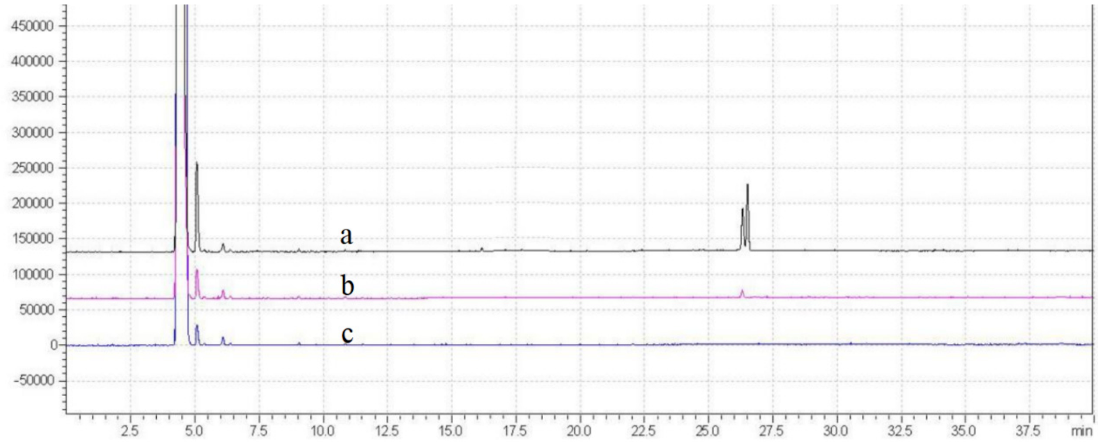


compound **6**
(a: D-L-rhamnose; b: compound **6**; c: blank)

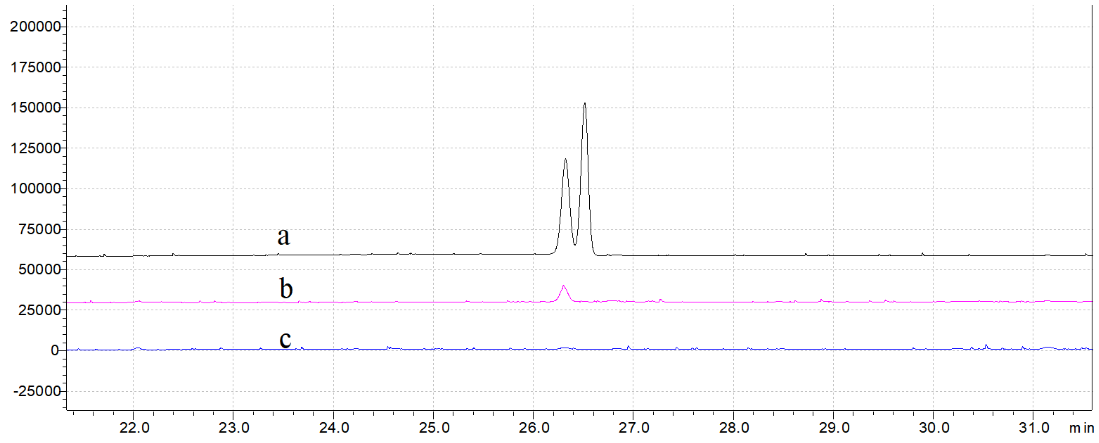


Extended compound **6**
(a: D-L-rhamnose; b: compound **6**; c: blank)

**Figure S51.** GC analysis of D-rhamnose, L-rhamnose and compounds **1–6**.
